# Transcriptomic analysis of adaptive mechanisms in response to sudden salinity drop in the mud crab, *Scylla paramamosain*

**DOI:** 10.1186/s12864-018-4803-x

**Published:** 2018-05-31

**Authors:** Huan Wang, Lei Tang, Hongling Wei, Junkai Lu, Changkao Mu, Chunlin Wang

**Affiliations:** 1 School of Marine Science, Ningbo University, Ningbo, 315211 Zhejiang China; 2 Key Laboratory of Applied Marine Biotechnology, Ministry of Education, Ningbo University, Ningbo, 315211 Zhejiang China

**Keywords:** *Scylla paramamosain*, Gill, Osmoregulation, Transcriptional profiling, Differentially expressed gene

## Abstract

**Background:**

*Scylla paramamosain* (Crustacea: Decapoda: Portunidae: Syclla De Hann) is a commercially important mud crab distributed along the coast of southern China and other Indo-Pacific countries (Lin Z, Hao M, Zhu D, et al, Comp Biochem Physiol B Biochem Mol Biol 208-209:29–37, 2017; Walton ME, Vay LL, Lebata JH, et al, Estuar Coast Shelf Sci 66(3–4):493–500, 2006; Wang Z, Sun B, Zhu F, Fish Shellfish Immunol 67:612–9, 2017). While *S. paramamosain* is a euryhaline species, a sudden drop in salinity induces a negative impact on growth, molting, and reproduction, and may even cause death. The mechanism of osmotic regulation of marine crustaceans has been recently under investigation. However, the mechanism of adapting to a sudden drop in salinity has not been reported.

**Methods:**

In this study, transcriptomics analysis was conducted on the gills of *S. paramamosain* to test its adaptive capabilities over 120 h with a sudden drop in salinity from 23 ‰ to 3 ‰.

**Results:**

At the level of transcription, 135 DEGs (108 up-regulated and 27 down-regulated) annotated by NCBI non-redundant (nr) protein database were screened. GO analysis showed that the catalytic activity category showed the most participating genes in the 24 s-tier GO terms, indicating that intracellular metabolic activities in *S. paramamosain* were enhanced. Of the 164 mapped KEGG pathways, seven of the top 20 pathways were closely related to regulation of the Na^+^ / K^+^ -ATPase. Seven additional amino acid metabolism-related pathways were also found, along with other important signaling pathways.

**Conclusion:**

Ion transport and amino acid metabolism were key factors in regulating the salinity adaptation of *S. paramamosain* in addition to several important signaling pathways.

**Electronic supplementary material:**

The online version of this article (10.1186/s12864-018-4803-x) contains supplementary material, which is available to authorized users.

## Background

Salinity as a key abiotic parameter that influences the distribution, abundance, physiology, and well-being of crustaceans. [3, 4, 15, 34]. Salinity is also an important factor in the production of crustacean aquaculture [47], which can affect growth, survival, molting, oogenesis, embryogenesis and larval quality [6, 14, 21, 30, 32, 35, 38]. The salinity of crustacean aquaculture can easily be affected by a local torrential rain [46]. Fortunately, most marine species which have been studied have osmoregulatory capacities to adjust to the shifting salinities of estuary and wetland waters within limits. In crustaceans, the adaptability of osmoregulation is primarily achieved by the gills [7, 12, 16, 28, 34, 36]. Since regulation of osmotic pressure in marine animals involves energy consumption, drastic changes in salinity can lead to death of the organism.

Recent studies have shown that low salinity influences ion channel activity [33, 37, 44, 45] and L-type free amino acids [1, 25, 39, 42, 43], which are tightly involved with osmoregulation. In particular, the Na^+^ / K^+^ -ATPase, a well-known ion channel, is the main ion transport enzyme of post-larvae in crustaceans. Its function in the organism is to enhance adaptability to salinity changes through osmoregulation [20, 22, 26, 27]. Chung and Lin [8] cloned the full-length α-subunit of the Na^+^ / K^+^ -ATPase cDNA, indicating the osmoregulatory role of the channel via both mRNA and protein expression. Likewise, Lu et al. [25] completed the cDNA cloning of glutamate dehydrogenase and its expression, indicating that GDH played an important role in controlling osmoregulation through free amino acids in *S. paramamosain*.

Although a great deal of progress had been made in the study of osmotic adjustment in crustaceans, there have been relatively fewer studies on *S. paramamosain*. In particular, the molecular mechanism of adaptation to sudden salinity drop has not yet been reported. Since *S. paramamosain* is a euryhaline species [40], it is easy to overlook the impact of salinity on the organism’s physiology. In the environment, a sudden drop in salinity caused by heavy rain over a short period of time (drop by > 10‰) may lead to death. In this study, we simulated a drastic reduction in salinity from 23‰ to 3‰. Then, the molecular mechanism of adapting to the salinity drop was analyzed by transcriptome analysis.

## Methods

### Experimental animals and sectionalization

A total of 300 randomly selected crabs with a body weight of ~ 30 g was selected and kept in a natural water environment with a salinity of 23 ‰ and a temperature of approximately 20 °C. Every 50 crabs were randomly selected (weight ~ 30 g) as a group, with a total of six groups, housed in six cement pools under identical physical and chemical conditions. The salinity of the seawater for three of the groups was adjusted to 3‰ from 23‰, which dropped by 20‰. These three groups were defined as the LS (low salinity) group. The other three groups were defined as the CK groups, where the salinity of seawater was kept at 23‰. All other conditions were the same as the LS group.

It should be noted that the space of a pond in the experiment was big enough for juvenile crabs, and we added a few tiles in ponds as shelter, which could effectively avoid fighting and killing each other.

### HE staining

Gill morphology and ultrastructure of *S. paramamosain* were observed using light microscopy after hematoxylin (HE) staining. HE staining was conducted according to the method of Wang et al. [41]. First, gills were set in paraffin and sliced into sections with a thickness of up to 5–10 μm. Then, the sections were de-waxed using xylene and rehydrated in an ethanol series. Sections were stained with eosin and HE which purchased from Invitrogen (Carlsbad, CA, USA). Preparation of 4% paraformaldehyde solution were made using filtered isotonic sea water: the salinity of 23‰ and 3‰ filtered sea water was used in the C and LS groups, respectively.

### Total RNA isolation and gene expression analysis

Total RNA was isolated from gill tissue using sqRT-PCR RNAiso Plus (TaKaRa, Dalian, China). The cDNA was synthesized using the Perfect Real Time version of the PrimerScriptTM RT reagent kit with gDNA Eraser (Perfect Real Time) (TaKaRa,) according to manufacturer’s instructions. Then, sq-RT-PCR were chosen to analyze genes, and performed in a total reaction volume of 25 μl according to the manufacturers’ instructions. The *S. paramamosain* beta-actin gene and 18S ribosomal RNA gene were selected as the internal control. Primers used in this study are listed in Additional file 1: Table S1.

### Transcriptome sequencing

For Illumina paired-end sequencing, equivalent quantities of total RNA isolated from the three mud crabs were pooled as one sample, and eventually there were three samples in each group, CK and LS. After poly (A) mRNA was purified and fragmented into small pieces, we used random hexamer primers and reverse transcriptase (Invitrogen) to carry out first-strand cDNA synthesis. Second-strand cDNA synthesis was performed with RNase H (Invitrogen) and DNA polymerase I (New England BioLabs, Beijing, China). A cDNA library was constructed with average insert sizes of 200–500 bp and cDNA sequencing was conducted using the Illumina HiSeqTM 4000 system according to the manufacturer’s protocols, with read length of 150 bp Transcriptome Quantification analyses two independent cDNA libraries were constructed for the two organs in parallel according to the Transcriptome protocol. The transcriptome sequencing was performed by BGI (BGI, Shenzhen, China).

### Analysis of differentially expressed genes

Because the annotated genome of *S. paramamosain* has not been published, de novo assembly was used here as reference for further analysis. Firstly, the raw reads were filtered to remove adaptor and low quality sequences. Afterfiltration, clean reads were assembled into unigenes using Trinity de novo assembler, followed by TGICL clustering tool. The reads from control (CK) and experimental group (LS) were mapped against the assembled Unigene using HISAT. The FPKM (fragments per kb per million reads) method was used to calculate the expression abundance. Each unigene was subjected to a BLASTX search against the NCBI non-redundant (nr) protein database with an e-value threshold of 10–3. R package DESeq2 were performed to identify the differentially expressed genes (DEGs). DEG was considered as unigene with greater than 2-fold change and *p*-value < 0.05. Gene ontology (GO) terms and KEGG pathway annotation were achieved using the Blast2GO program and kaas (KEGG Automatic Annotation Server) on-line program (http://www.genome.jp/kaas-bin/kaas_main), respectively.

## Results

### Adaptive phenotype of *S. paramamosain* to sudden drop in salinity

**Fig. 1:**
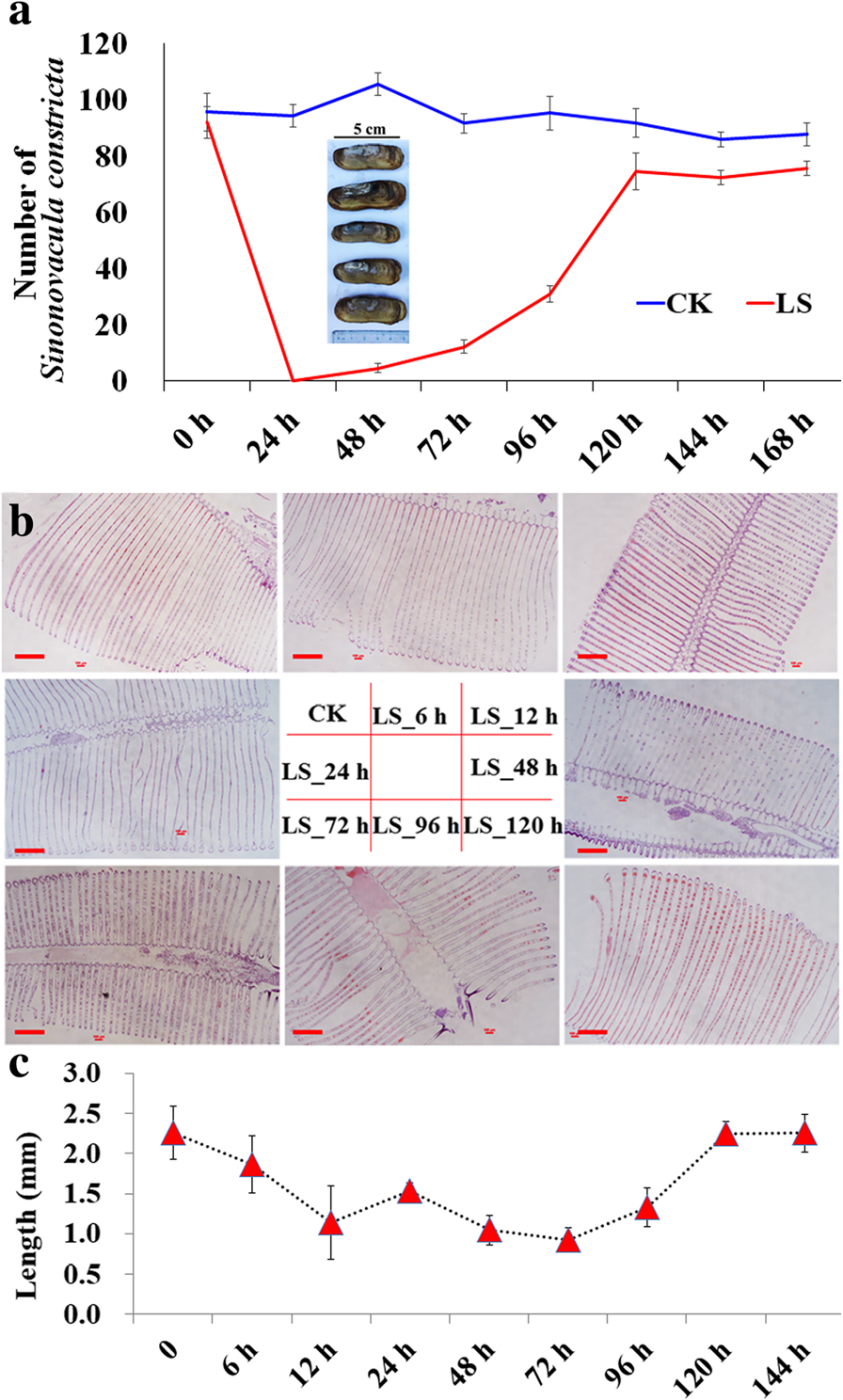
Food intake and morphology change of gills in response to the sudden drop in salinity in *S. paramamosain*. **a**, Food intake change in *Scylla paramamosain*. The juvenile crab with a body weight of ~ 30 g eat about two *Sinonovacula constricta* (Lamarck) in 24 h, so three *Sinonovacula constricta* (Lamarck) per crab were feed every day and cleaned up the leftovers regularly to prevent the water going bad. **b**, the phenotypic changes of gill filaments within 120 h after the salinity drop: 23‰ in CK group, others were the phenotypes of 12, 24, 48, 72, 96, and 120 h after salinity dropped to 3‰. **c**, changes of gills in length; “0” indicated CK group, and the others were the gill length at 12, 24, 48, 72, 96, 120 h after salinity dropped to 3‰. Bars are 300 μm in **b**

There were four deaths in the CK group within 7 days and 24 deaths in the LS group. The LS death time was concentrated within 24, 48, and 72 h. In addition, the LS group showed hyperactivity within 48 h. As time went by, the motility was diminished and normalized (Fig. 1a). The LS group did not have food over 72 h, and gradually started to eat over time. Conditions returned to normal after 120 h.

The gills are an important organ in osmoregulation of marine crustaceans, with the gill filament serving as the basic units of function. According to the results of gill slicing, under normal conditions, the gill filaments of the crab in the CK group were regular (Fig. 1b). Gills of crabs in the LS group became shorter and thicker from 6 h and reached the shortest at 72 h, which was less than half that of gills observed in the CK group (Fig. 1a & b). Gills then gradually became longer with time and returned to normal after 120 h (Fig. 1b & c). The changes in gill filament anatomy was consistent with those of the physiological activities mentioned above, suggesting that changes in gill filaments play an important regulatory role in adaptation of *S. paramamosain* to decrease in salinity.

### Differentially expressed genes (DEGs) in the gill of *S. paramamosain*

Gills of marine crustaceans play an important role in osmoregulation. In our study, the mud crabs had adjusted to a salinity of 3‰ in 120 h after a sudden drop in salinity. In order to study the molecular mechanism underlying this adaptation, we performed transcriptional profiling at 120 h.

Approximately 39.6 Gb bases were generated in total on the BGISEQ-500 sequencing platform. Because the genome sequencing of *S. paramamosain* has not yet been elucidated, after reads filtering, Trinity [18] was used to perform de novo assembly with clean reads (Additional file 1: Table S2). Tgicl ([31] Pertea et al., 2003) was used on cluster transcripts to remove abundance and get Unigenes (Additional file 1: Table S3). The proportion of bases with low quality (< 20) was very low in all samples, indicating a high quality of sequences. Finally, high-quality transcripts were obtained (Table 1) and used as reference sequences. Genes were annotated using Unigenes by aligning with seven functional database as follows 36,376 (NR: 34.59%), 38,958 (NT: 37.04%), 26,425 (Swissprot: 25.13%), 26,056 (KOG: 24.77%), 28,890 (KEGG: 27.47%), 4859(GO: 4.62%), and 19,756 (InterPro: 18.78%) (Additional file 1: Figure S2). For functional annotation results, we detected 32,627 CDS by Transdecoder. We also detected 74,041 SSR distributed on 44,075 unigenes, and predicted 12,623 transcription factor (TF) coding unigenes.

**Table 1:** Quality metrics of transcripts in the gill of *Scylla paramamosain*

Sample	Total Number	Total Length (bp)	Mean Length	N50	N70	N90	GC(%)
CK_1	93,877	54,675,298	582	952	445	232	45.50
CK_2	100,075	57,680,722	576	938	436	230	45.61
CK_3	97,185	52,573,783	540	803	400	225	45.75
LS_1	87,582	49,398,653	564	885	424	229	45.75
LS_2	97,564	51,685,899	529	777	390	222	45.88
LS_3	81,111	58,440,218	720	1354	617	262	46.89

Sample: Sample name; Total Number: The total number of transcripts; Total Length: The read length of transcripts; Mean Length: The average length of transcripts; N50: The N50 length was used to determine the assembly continuity such that the higher the better. N50 is a weighted median statistic such that 50% of the total length is contained in the unigenes that are equal to or larger than this value. N70: Similar to the N50; N90: Similar to the N50. GC (%): the percentage of G and C bases in all transcripts

**Fig. 2:**
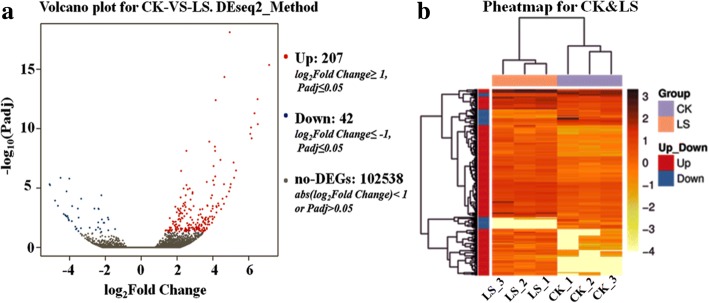
Differentially expressed genes (DEGs) from the gill of *S. paramamosain*. **a**, Volcano plot of DEGs; **b**, Heatmap of DEGs. Fold change> = 2.00 and adjusted p value <= 0.05

A total of 249 genes was differentially expressed in the LS Group and the CK group, including 207 up-regulated genes and 42 down-regulated genes (fold change> = 2.00 and adjusted *p* value <= 0.05) (Fig. 2). Of the 249 DEGs, 217 were annotated at least one of the following: Nr (192), Nt (160), Swissprot (154), KEGG (163), KOG (152), Interpro (130) and GO (32. No description was found for 3 DEGs (3 of which are down-regulated genes). Of the 192 differential genes annotated in the Nr database, 57 were described as “hypothetical” in the GenBank database and were also excluded from further analysis. Finally, 135 DEGs that were annotated with Nr were screened, out of which 108 were up-regulated and 27 were down-regulated genes (Additional file 1: Table S4).

**Fig. 3:**
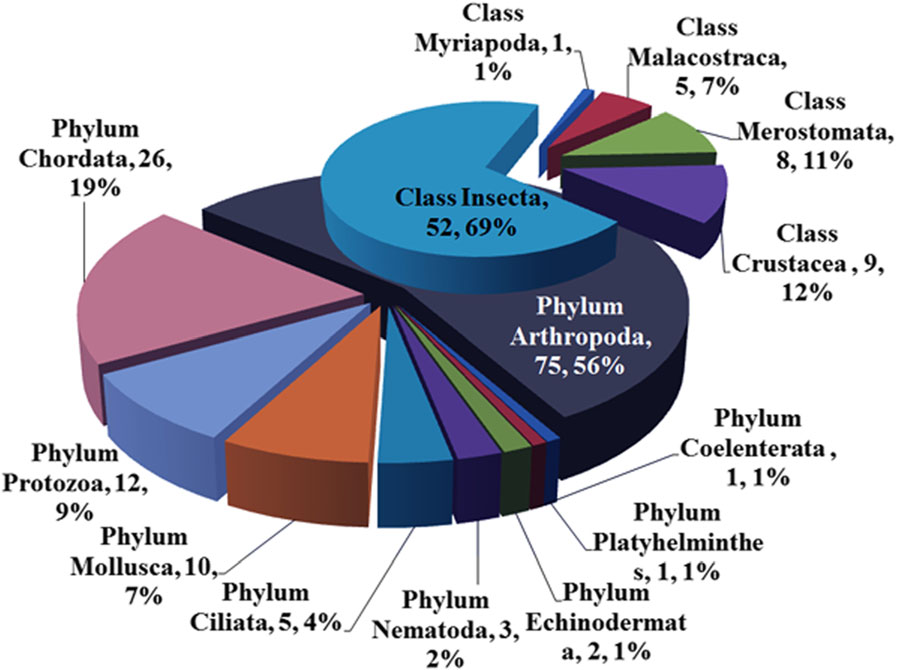
Class distribution diagram of annotations form Nr database of 135 differentially expressed genes (DEGs) in response to the sudden drop in salinity in the gill of *S. paramamosain*. These 135 DEGs showed a certain degree of similarity to genes belonging to phylum Arthropoda (75 DEGs), Chordata (26), Protozoa (12), Mollusca (10), Ciliata (5), Nematoda (3), Echinodermata (2), Coelenterata (1), and Platyhelminthes (1 DEG). The pie chart on the lower side of the image shows taxonomic groups of Phylum, and each fan represented a phylum. The upper pie chart showed taxonomic groups of Class which contain six classes. Numbers and proportion of DEGs are counted in each end of the bar

Of these 135 DEGs, BUD13 homolog (ID: CL3353.Contig2) was the highest-upregulated gene (90.13 fold) followed by serine/threonine-protein kinase (ID: CL90.Contig3, 71.65 fold) and beta-1, 4-*N*-acetylgalactosaminyl transferase (ID: CL2951.Contig2; 67.99 fold) (Additional file 1: Table S4). The three most highly down-regulated genes were uncharacterized proteins LOC107039269 (ID: CL94.Contig5; − 34.61 fold), kinesin light chain (ID: CL643.Contig10; − 34.03 fold) and aquaporin-12 (ID: CL5376.Contig4; − 27.99 fold) (Additional file 1: Table S4). 75 (56%) showed the highest similarity to genes belonging to phylum Arthropoda (Fig. 3). Of the 75 DEGs, 52 showed highest similarity to genes from class Insecta, and the remainder to genes from class Crustacea (9 DEGs), Merostomata (8 DEGs), Malacostraca (5 DEGs), and Myriapoda (1 DEG) (Fig. 3). The remaining 60 DEGs (44%) belonged to the Phylum Chordata (26 DEGs), Protozoa (12 DEGs), Mollusca (10 DEGs), Ciliata (5 DEGs), Nematoda (3 DEGs), Echinodermata (2 DEG), Coelenterata (1 DEG), and Platyhelminthes (1 DEG) (Fig. 3).

### Functional annotation

**Fig. 4:**
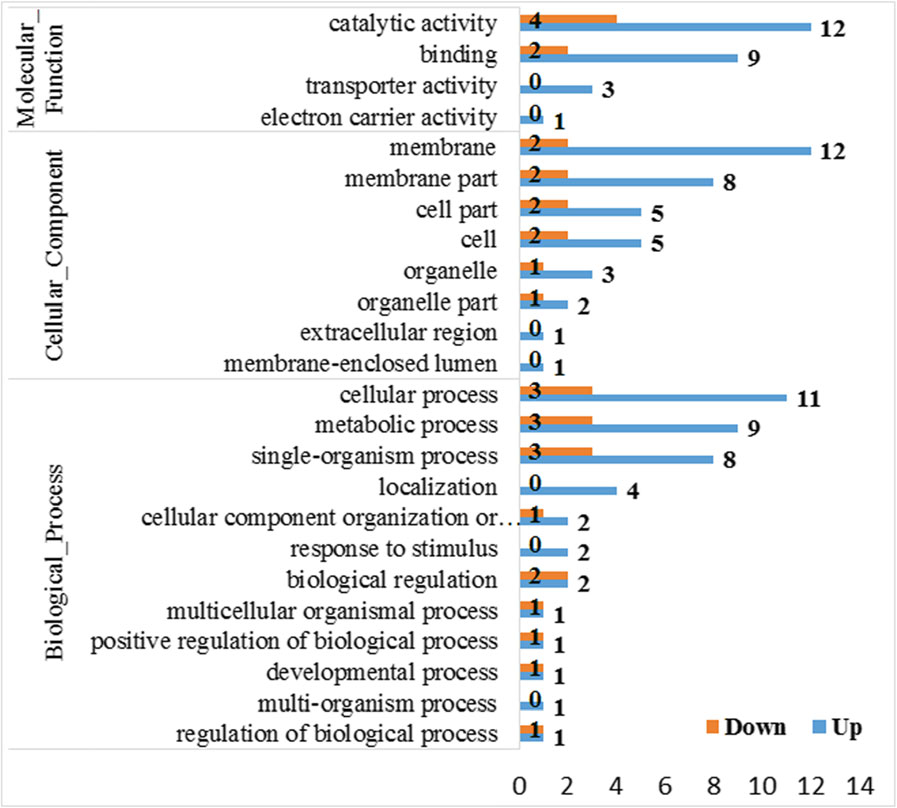
GO annotation of differentially expressed genes (DEGs) in response to the sudden drop in salinity in the gill of *S. paramamosain*. DEGs were assigned to second-tier GO categories associated with three parent terms: biological process, cellular component, and molecular function. Blue bars indicate up-regulated DEGs and orange bars indicate down-regulated DEGs. Numbers of DEGs are counted in each end of bar

Gene Ontology (GO) analysis was performed on DEGs using Blast2GO [9, 17]. According to the second-tier GO terms, 32 DEGs were assigned 24 GO annotations which represented three main GO categories: biological process (18), cellular component (18), and molecular function (23) (Fig. 4 & Additional file 1: Figure S3). Among them, 24 GO terms were assigned to the up-regulated group and 17 to the down-regulated group. Twelve processes were identified in the biological process category, with 14 DEGs involved in cellular processes, 12 involved in metabolic processes, and 11 involved in single-organism processes. This identified three biological processes as the most strongly affected in the gill of *S. paramamosain* by the sudden drop in salinity (Fig. 4). In the cellular component category, membrane (14), membrane part (10), cell parts (7), and cell (7) were most involved (Fig. 4). In the molecular function category, only four items contained the catalytic activity (16), binding (11), transporter activity (3) and electron carrier activity (1) (Fig. 4). It is worth noting that the catalytic activity category (14) had the most participating genes in the 24 s-tier GO terms. The results suggested that the salinity of the water environment dropped sharply. In order to maintain life activity, the metabolic activity of the cells in *S. paramamosain* was strengthened, in particular the catalytic function of enzymes regulating ion changes and osmotic pressure, processes involved in adapting to the new environment with dropped salinity.

In addition, among all the 24 s-tier GO terms, localization, response to stimulus, and multi-organization processes from the biological process category, as well as the extracellular region, membrane-enclosed lumen from cellular component, transporter activity, and electron carrier activity from molecular function categories were all upregulated DEGs with no downregulated DEGs.

### DEGs pathway analysis

**Fig. 5:**
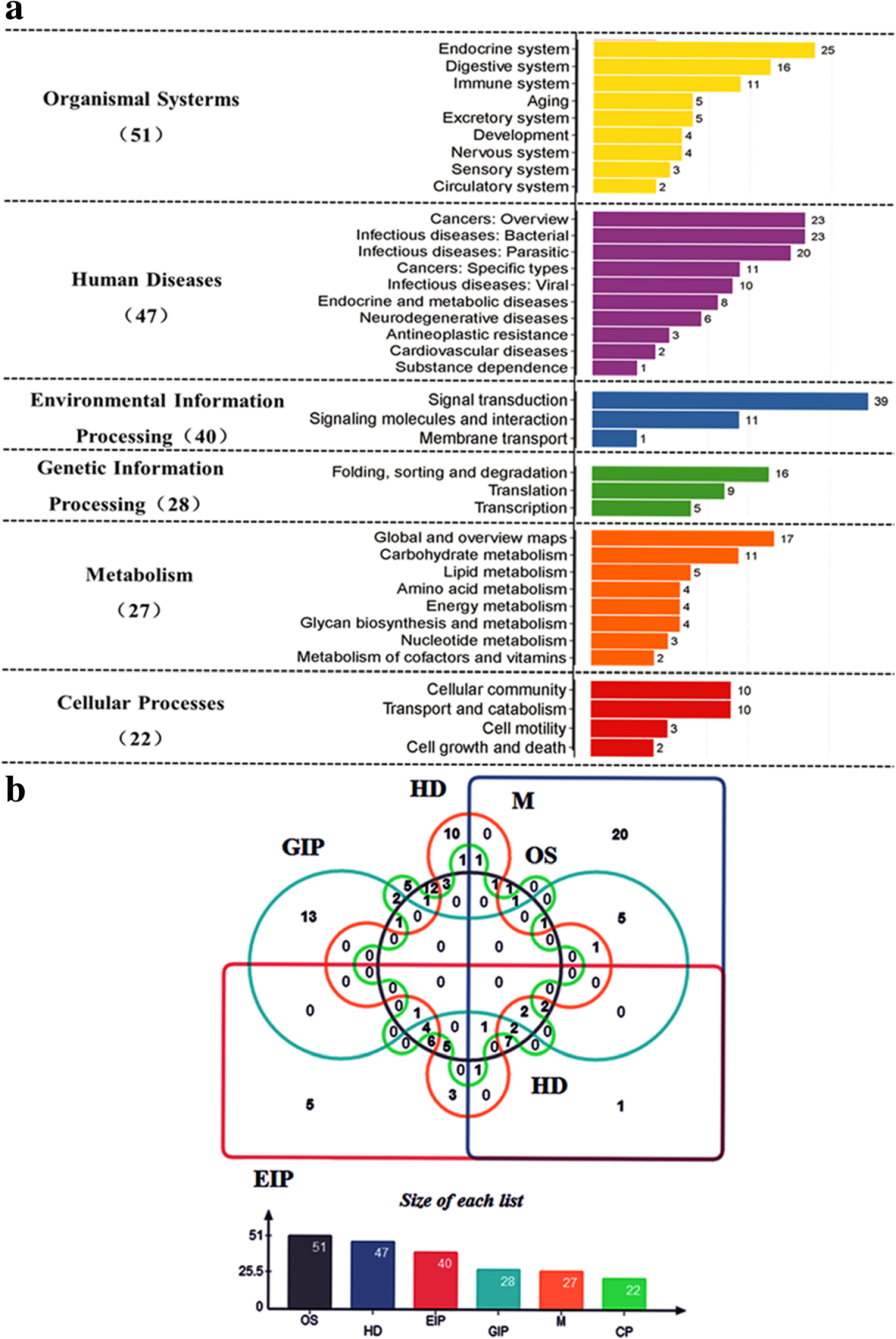
Pathway classification (**a**) and differentially expressed genes (DEGs) distribution (**b**). B, OS: Organismal Systems, HD: Human Disease, EIP: Environmental Information Processing, GIP: Genetic Information Processing, M: Metabolism, CP: Cellular Processes. X axis represents the number of DEGs. Y axis represented functional classification of KEGG. There were seven branches for KEGG pathways: Cellular Processes, Environmental Information Processing, Genetic Information Processing, Human Disease (For animals only), Metabolism, Organismal Systems and Drug Develop (all of the DEGs did not involve)

With DEGs, KEGG pathway classification was performed (Fig. 5) according to the KEGG database website (http://www.genome.jp/kegg/pathway.html). Of all DEGs, 119 were mapped in 164 KEGG pathways which were graded into six categories (level 1) according to their biological function, including organismal systems (51), human diseases (47), environmental information processing (40), genetic information processing (28), metabolism (27), cellular processes (22) (Fig. 5a & b).

Of the 164 pathways, 51 (42.86%) were related to the organismal systems category which was subdivided into nine subsets (level 2): aging, circulatory system, development, digestive system, endocrine system, excretory system, immune system, nervous system, sensory system (Fig. 5a and Table 2). The detailed KEGG pathways of organismal systems and DEGs involved are presented in Table 2. 47 (39.50%) were related to human diseases (Fig. 5a & b). These genes contained Unigene45614 (toxoplasmosis, ko05145) annotated cytochrome C (*Marsupenaeus japonicus*) CL979. Contig1 (insulin resistance, ko04931) annotated solute carrier family 2, Unigene 44,862 (*Staphylococcus aureus* infection, ko05150) annotated keratin, type I cytoskeletal 9 (*Marmota marmota marmota*), CL979. Contig1 (pathways in cancer, ko05200) annotated solute carrier family 2, facilitated glucose transporter member 3 (*Zootermopsis nevadensis*) (Additional file 1: Table S5). It is worth mentioning, these genes were not disease genes, but they were relevant in certain pathologies.

**Table 2:** Organismal Systems pathways and DEGs involved

Pathway	DEGs genes (51)	Pathway ID	Level 2
Longevity regulating pathway - multiple species	Unigene32292, Unigene35344, Unigene35501	ko04213	Aging
Longevity regulating pathway	Unigene32292, Unigene35501, CL3443.Contig1	ko04211	Aging
Longevity regulating pathway - worm	CL3933.Contig2	ko04212	Aging
Cardiac muscle contraction	Unigene11331	ko04260	Circulatory system
Adrenergic signaling in cardiomyocytes	Unigene11331, Unigene44161	ko04261	Circulatory system
Vascular smooth muscle contraction	Unigene44161	ko04270	Circulatory system
Osteoclast differentiation	Unigene19465, Unigene31660	ko04380	Development
Axon guidance	Unigene40814	ko04360	Development
Dorso-ventral axis formation	Unigene34154	ko04320	Development
Protein digestion and absorption	Unigene12705, CL6154.Contig2, Unigene38622, CL922.Contig5, Unigene11331, CL922.Contig9, CL452.Contig3, CL922.Contig7, Unigene17070, CL3933.Contig2	ko04974	Digestive system
Bile secretion	Unigene12705, Unigene33785, Unigene38622, Unigene11331, CL979.Contig3, CL979.Contig1, Unigene17070	ko04976	Digestive system
Mineral absorption	Unigene12705, Unigene15443, Unigene38622, Unigene11331, Unigene17070	ko04978	Digestive system
Carbohydrate digestion and absorption	Unigene11331	ko04973	Digestive system
Vitamin digestion and absorption	CL4600.Contig3	ko04977	Digestive system
Salivary secretion	Unigene10578, Unigene11331	ko04970	Digestive system
Gastric acid secretion	Unigene11331	ko04971	Digestive system
Pancreatic secretion	Unigene11331	ko04972	Digestive system
Thyroid hormone signaling pathway	Unigene33785, Unigene111, Unigene113, Unigene41655, Unigene36212, Unigene31721, Unigene11331, Unigene112, CL979.Contig3, CL979.Contig1, Unigene17270	ko04919	Endocrine system
Adipocytokine signaling pathway	Unigene33785, CL3443.Contig1, CL979.Contig3, CL979.Contig1	ko04920	Endocrine system
Insulin secretion	Unigene33785, Unigene11331, CL979.Contig3, CL979.Contig1, CL5287.Contig1	ko04911	Endocrine system
Thyroid hormone synthesis	Unigene10157, Unigene27634, Unigene11857, Unigene11331	ko04918	Endocrine system
PPAR signaling pathway	CL1683.Contig3, CL1683.Contig5, CL2638.Contig2	ko03320	Endocrine system
Estrogen signaling pathway	Unigene10157, Unigene35344, Unigene27634	ko04915	Endocrine system
Progesterone-mediated oocyte maturation	Unigene10157, Unigene27634	ko04914	Endocrine system
Glucagon signaling pathway	Unigene33785, CL979.Contig3, CL979.Contig1	ko04922	Endocrine system
Renin-angiotensin system	Unigene49236	ko04614	Endocrine system
Insulin signaling pathway	Unigene32292, Unigene35501, Unigene44161	ko04910	Endocrine system
Oxytocin signaling pathway	Unigene44161	ko04921	Endocrine system
GnRH signaling pathway	CL6599.Contig1	ko04912	Endocrine system
Proximal tubule bicarbonate reclamation	Unigene12705, Unigene15693, Unigene38622, Unigene11331, Unigene17070	ko04964	Excretory system
Aldosterone-regulated sodium reabsorption	Unigene11331	ko04960	Excretory system
Endocrine and other factor-regulated calcium reabsorption	Unigene11331	ko04961	Excretory system
Antigen processing and presentation	Unigene10157, Unigene35344, Unigene27634, Unigene11857, Unigene41750	ko04612	Immune system
NOD-like receptor signaling pathway	Unigene10157, Unigene27634	ko04621	Immune system
Hematopoietic cell lineage	Unigene15456, Unigene47891l, Unigene15457	ko04640	Immune system
B cell receptor signaling pathway	Unigene15456, Unigene47891, Unigene15457	ko04662	Immune system
Platelet activation	Unigene44161	ko04611	Immune system
Fc gamma R-mediated phagocytosis	CL6599.Contig1, CL4852.Contig1	ko04666	Immune system
Long-term potentiation	Unigene44161	ko04720	Nervous system
GABAergic synapse	CL3399.Contig2	ko04727	Nervous system
Dopaminergic synapse	Unigene44161	ko04728	Nervous system
Glutamatergic synapse	CL6599.Contig1, Unigene5252, CL3399.Contig2	ko04724	Nervous system
Phototransduction	CL6217.Contig3, CL41.Contig2	ko04744	Sensory system
Olfactory transduction	CL6217.Contig3, CL41.Contig2	ko04740	Sensory system
Inflammatory mediator regulation of TRP channels	Unigene44161	ko04750	Sensory system

Forty genes (33.61%) were related to environment information processing which was subdivided into three subsets: signal transduction, signaling molecules and interaction, and membrane transport. Of 164 pathways, 39 genes were most abundant in the signal transduction category, which contained 20 KEGG pathways (Additional file 1: Table S6): AMPK signaling pathway (ko04152), PI3K-Akt signaling pathway (ko04151), HIF-1 signaling pathway (ko04066), cGMP-PKG signaling pathway (ko04022), phosphatidylinositol signaling system (ko04070), hedgehog signaling pathway in fly (ko04341), hedgehog signaling pathway (ko04340), sphingolipid signaling pathway (ko04071), cAMP signaling pathway (ko04024), notch signaling pathway (ko04330), MAPK signaling pathway in fly (ko04013), hippo signaling pathway (ko04390), FoxO signaling pathway (ko04068), MAPK signaling pathway (ko04010), calcium signaling pathway (ko04020), Wnt signaling pathway (ko04310), mTOR signaling pathway (ko04150), hippo signaling pathway in fly (ko04391), Ras signaling pathway (ko04014), and phospholipase D signaling pathway (ko04072). In addition, the other pathways of environment information processing were involved, such as ABC transporters (ko02010) which belonged to membrane transport, ECM-receptor interaction (ko04512), cell adhesion molecules (CAMs) (ko04514), and neuroactive ligand-receptor interaction (ko04080), which belonged to signaling molecules and interaction. Throughout all of environment information processing pathways, the results indicated gill as an important organ for salinity regulation for *S. paramamosain.* After the gills detected the salinity drop, the salinity drop transformed into stress signals transmitted to other parts of the body, thereby initiating regulation mechanism used by the gills. A complex molecular signal feedback mechanism for regulation could ultimately achieve osmotic pressure balance of as an adaptive response to a sudden salinity drop.

**Fig. 6:**
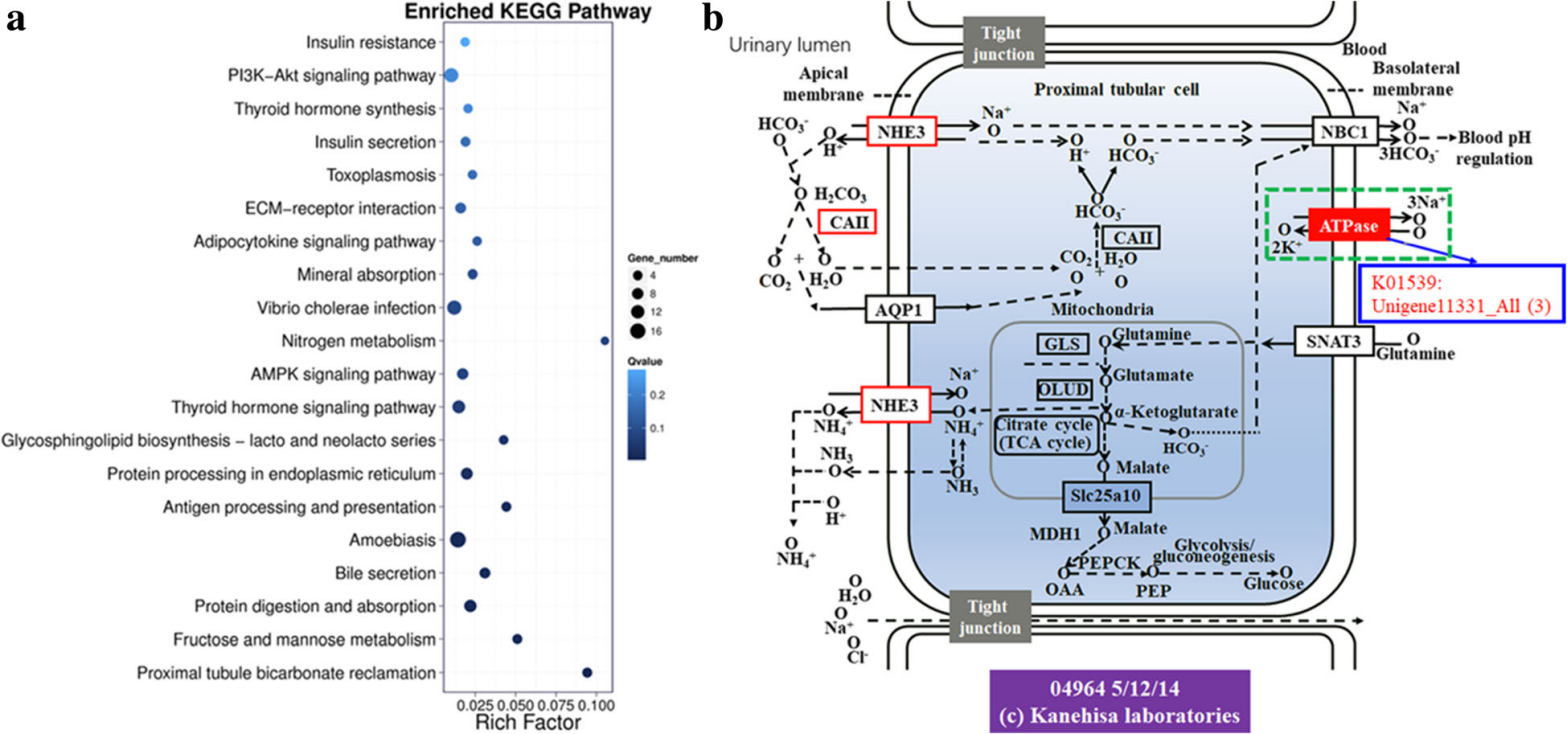
Pathway functional enrichment of differentially expressed genes (DEGs) (**a**) and pathway of Proximal tubule bicarbonate reclamation (ko04964)(**b**). A, X axis represents enrichment factor. Y axis represents pathway name. The color indicates the q value (high: white, low: blue), the lower q value indicates the more significant enrichment. Point size indicates DEG number (The larger dots refer to larger amount). Rich Factor refers to the value of enrichment factor, which is the quotient of foreground value (the number of DEGs) and background value (total Gene amount). The larger the value, the more significant the enrichment. **b**, Pathways were mapped using the KEGG Mapper (http://www.genome.jp/kegg/mapper.html). Up-regulated DEGs are boxed in red, DEG ID numbers are shown outside the box in blue

Functional enrichment was also performed on DEGs according to the above KEGG pathway classification. The top 20 pathways (Fig. 6a & Table 3) showed that seven pathways were directly related to the active regulation of the Na^+^ / K^+^-ATPase enzyme: Proximal tubule bicarbonate reclamation (ko04964), protein digestion and absorption (ko04974), bile secretion (ko04976), thyroid hormone signaling pathway (ko04919), mineral absorption (ko04978), insulin secretion (ko04911), thyroid hormone synthesis (ko0491), and the regulatory genes of Na^+^ / K^+^-ATPase were all up-regulated。Crustacean Na^+^ / K^+^ -ATPase plays an important role in the regulation of hematopoietic osmotic pressure at different salinities [2]. Current research on crustacean Na^+^ / K^+^ -ATPase has been extensively reported [10, 23] suggesting that it is a widespread P-type ATPase that plays an important role in maintaining Na^+^, K^+^ homeostasis [11]. As all known, free amino acids [1, 25, 39, 42, 43] also played an important role in osmoregulation. In addition, several pathways connected with amino acid metabolism were detected, such as for arginine biosynthesis (ko00220) (Additional file 1: Table S8), alanine, aspartate and glutamate metabolism (ko00250) (Additional file 1: Table S8), lysine degradation (ko00310) (Additional file 1: Table S8), valine, leucine and isoleucine degradation (ko00280) (Additional file 1: Table S8), amino sugar and nucleotide sugar metabolism (ko00520) (Additional file 1: Table S8), biosynthesis of amino acids (ko01230) (Additional file 1: Table S8), pyrimidine metabolism (ko00240) (Additional file 1: Table S8). In addition, several important signal pathways were found, cAMP signaling pathway (ko04024) (Additional file 1: Table S6), MAPK signaling pathway (ko04013, ko04010) (Additional file 1: Table S6), Wnt signaling pathway (ko04310) (Additional file 1: Table S6), mTOR signaling pathway (ko04150) (Additional file 1: Table S6), Ras signaling pathway (ko04014) (Additional file 1: Table S6). The results implied these pathways might all take part in osmoregulation.

**Table 3:** The greatest functional classification differences between CK and LS

NO	Pathway	DEGs genes (119) / All genes (28890) with pathway annotation	*P* value	Pathway ID
1	Proximal tubule bicarbonate reclamation	4 (4.2%) / 53 (0.18%)	1.239607e-05	ko04964
2	Fructose and mannose metabolism	5 (4.2%) / 98 (0.34%)	0.000239203	ko00051
3	Protein digestion and absorption	10 (8.4%) / 456 (1.58%)	0.000280083	ko04974
4	Bile secretion	7 (5.9%) / 226 (0.78%)	0.0003098698	ko04976
5	Amoebiasis	17 (14.3%) / 1195 (4.14%)	0.0004429313	ko05146
6	Antigen processing and presentation	5 (4.2%) / 113 (0.39%)	0.0004620034	ko04612
7	Protein processing in endoplasmic reticulum	9 (7.6%) / 455 (1.57%)	0.001166426	ko04141
8	Glycosphingolipid biosynthesis - lacto and neolacto series	4 (3.4%) / 94 (0.33%)	0.002007803	ko00601
9	Thyroid hormone signaling pathway	11 (9.2%) / 746 (2.58%)	0.003509184	ko04919
10	AMPK signaling pathway	8 (6.7%) / 465 (1.61%)	0.005048467	ko04152
11	Nitrogen metabolism	2 (1.7%) / 19 (0.07%)	0.005079646	ko00910
12	Vibrio cholerae infection	14 (11.8%) / 1170 (4.05%)	0.006652947	ko05110
13	Mineral absorption	5 (4.2%) / 214 (0.74%)	0.007464861	ko04978
14	Adipocytokine signaling pathway	4 (3.4%) / 153 (0.53%)	0.01119608	ko04920
15	ECM-receptor interaction	7 (5.9%) / 440 (1.52%)	0.01272333	ko04512
16	Toxoplasmosis	4 (3.4%) / 172 (0.6%)	0.01655378	ko05145
17	Insulin secretion	5 (4.2%) / 264 (0.91%)	0.01723485	ko04911
18	Thyroid hormone synthesis	4 (3.4%) / 195 (0.67%)	0.02489002	ko04918
19	PI3K-Akt signaling pathway	14 (11.8%) / 1386 (4.8%)	0.0255091	ko04151
20	Insulin resistance	4 (3.4%) /214 (0.74%)	0.0333897	ko04931

### Validity of DEGs in transcriptomic data

Ten differentially expressed genes were randomly sampled for verification by transcriptional level experiments, and the results were consistent with those of DEG analyses (Table 4 & Additional file 1: Figure S3), indicating that the results of DEG were reliable.

**Table 4:** Validity of DEGs in Transcriptomic data

Gene ID	Transcriptome results	verification results
Gene.7653	up	up
Gene.16162	up	up
Gene.54888	up	up
Gene.22643	up	up
Gene.6925	up	up
Gene. 803	down	down
Gene. 18,132	down	down
Gene. 1196	down	down
Gene. 397	down	down
Gene. 2748	down	down

Gene. 7653: CL1096.Contig1_All, Gene. 16,162: CL2951.Contig2_All, Gene. 54,888: Unigene41750_All, Gene. 22,643: CL4861.Contig2_All, Gene. 6925: CL979.Contig3_All, Gene. 803: CL94.Contig5_All, Gene. 18,132: CL3482.Contig1_All, Gene. 1196:CL4395.Contig1_All, Gene. 397: CL41.Contig2_All, Gene. 2748: CL358.Contig1_All. The *S. paramamosain* beta-actin gene and 18S rRNA gene were selected as the internal control

## Discussion

The mechanism of osmotic regulation of aquatic crustaceans has received attention in recent years. Extensive work and quite a few important results have been reported on the morphological structure of osmotic regulatory organs [5, 24], ion transport regulation [10, 23, 33], regulation of hemolymph osmoregulation ([19];Huong et al., 2001, [42]), and neuroendocrine regulation [13, 29]. For both euryhaline and stenohaline species, changes in salinity will result in the organism adapting through the regulation of the neuroendocrine system, osmotic regulatory organs (mainly gills), hematopoietic osmotic pressure, and ion transport. A series of changes will occur to adapt to the changing external environment in order to maintain normal physiological and metabolic activity. However, few studies on neuroendocrine regulation, regulation of ion transport enzymes, or osmotic regulation are reported for *S. paramamosain*. Thus, this paper focuses on osmotic regulation in aquatic crustaceans. Since sequencing of the genome of *S. paramamosain* has not been completed yet, much of the information obtained still depends on transcriptomics.

*S. paramamosain* is a euryhaline species, and especially loves living in shallow sea and estuary nearshore. In China, the salinity of seawater in *S. paramamosain* ponds on the farm is between 25‰ and 3‰ in most area, and the minority such as in Shanghai is below 3‰. There is a production experience in the actual production process that amplitude of variation in salinity exceeding 10‰ would cause death for mud crabs (This only refers to sudden salinity drop). 23‰ is a normal salinity of the seawater for juvenile crabs S. paramamosain, which was living in the salinity before our treatment. We had made a preliminary experiment to select a salinity for treatment with 10 juvenile crabs as a group, including 13‰, 8‰, 5‰, 3‰, and 1‰, and the degree of salinity drop was 10‰, 15‰, 18‰, 20‰, and 22‰, respectively. In the end, we found some individuals in salinity 3‰ begin to die, and most individuals nearly died in salinity 1‰. So 3‰ might be the optimal choice as a critical point for the research of adaptive mechanism responding to sudden drop in salinity, and we finally selected the 3‰ in our study. Moreover, the LS death time was concentrated within 24, 48, and 72 h. The LS group showed hyperactivity within 48 h, and as time went by, the motility was diminished and normalized. The LS group did not have food over 72 h, and gradually started to eat over time. Conditions returned to normal after 120 h. To be honest, the mechanism of adaptive process is very complex, and need more in-depth research in the future work. The study aimed at the adaptive mechanism in response to sudden salinity drop. So we compared the CK with 120 h_group (a state of complete adaptation) to discover the difference of between normal condition and the adaptive status after sudden salinity drop from 23‰ to 3‰, and eventually reveal the adaptive mechanisms in response to sudden salinity drop in the mud crab, S. paramamosain at the level of transcription.

To date, reports on the regulation of infiltration of *S. paramamosain* are still quite limited to cloning and expression of the Na^+^ / K^+^ -ATPase [8] and cloning and expression of glutamate dehydrogenase (GDH) [25]. GHD is an important enzyme for the metabolism of glycine, proline, and alanine, which serve as general osmolytes in aquatic animals. In this study, GO annotation of DEG analysis showed that the most involved genes were derived from the category of catalytic activity of molecular function (Fig. 4), suggesting that the possible role of free amino acids in osmotic regulation is by enzymolysis. In addition, we found that Na^+^ / K^+^-ATPase is very active through the KEGG pathway classification and functional enrichment of DEGs (Figs. 5 and 6). The results showed that the Na^+^ / K^+^ -ATPase strengthened the ion exchange function necessary to maintain the osmotic balance required for normal survival after salinity drop from 23‰ to 3‰. In addition, there were many other KEGG pathways and differentially expressed genes, which might directly or indirectly participate in the regulation of osmotic adjustment of *S. paramamosain*, providing a valuable data source for subsequent studies.

*S. paramamosain* normally lives in estuary areas, where the environment is significantly different from freshwater, brackish water and seawater. Sometimes the salinity changes constantly, and this change requires a response in behavior, morphology, and biochemical physiology. In production, the sudden drop in salinity usually results from heavy rainfall in strong convective weather. For example, two typhoons hit Zhuhai in August 2017 in one week in Guangdong Province, causing huge losses to the crab farming industry. In this study, the cumulative salvage rate in six days was 16% (24/150) in the salinity sag test but may be higher in actual production. Because of the more complicated water environment system in production, the sudden drop in salinity is often accompanied by a decrease in water temperature. The comprehensive factors led to a decrease in crab immunity. As a result, there was an increase in pathogenic microorganisms in the water and an increase in crab mortality. In this study, only the salinity was changed and the rest of the environmental factors were controlled. For the first time, the molecular mechanism of *S. paramamosain* adapting to the salinity drop was studied. Through the research presented here, we had discovered a large number of potential genes that are related to the salinity adaptation in *S. paramamosain*. The possible KEGG pathway provided a basis for further research. In addition, this study was an important supplement to the physiological study of aquatic crustacean infiltration, but also provided a scientific basis for the regulation of crustacean aquaculture.

## Conclusions

In conclusion, we analyzed transcriptomic changes in the gills after a sudden drop in salinity in *S. paramamosain*. One hundred thirty-five DEGs annotated by Nr were screened, of which 108 were up-regulated and 27 were down-regulated. GO analysis showed that catalytic activity (14) had the most participating genes in the 24 s-tier GO terms, indicating that intracellular metabolic activities in *S. paramamosain* were enhanced. Based on KEGG pathway and biological functional enrichment on DEGs, the top 20 pathways showed that seven pathways were directly related to the active regulation of the Na^+^/K^+^ATP enzyme: Proximal tubule bicarbonate reclamation (ko04964), protein digestion and absorption (ko04974), bile secretion (ko04976), thyroid hormone signaling pathway (ko04919), mineral absorption (ko04978), Insulin secretion (ko04911), thyroid hormone synthesis (ko0491), and the regulatory genes in Na^+^/K^+^ATPase were all up-regulated. Additionally, several amino acid metabolism pathways were detected: arginine biosynthesis (ko00220), alanine, aspartate and glutamate metabolism (ko00250), lysine degradation (ko00310), valine, leucine and isoleucine degradation (ko00280), amino sugar and nucleotide sugar metabolism (ko00520), biosynthesis of amino acids (ko01230), pyrimidine metabolism (ko00240). In addition, some famous signal pathways were found, such as cAMP signaling pathway (ko04024), MAPK signaling pathway (ko04013, ko04010), Wnt signaling pathway (ko04310), mTOR signaling pathway (ko04150), Ras signaling pathway (ko04014). Our findings suggest that not only Na^+^/K^+^ATPase and amino acids played a key role in osmoregulation, but also some important signal pathways participated in osmoregulation. Ultimately, survival of *S. paramamosain* may be sustained in new surroundings with a sudden drop in salinity (23‰ fell to 3‰), by adjusting to the low salinity. The functional genomic studies of DEGs obtained in this study allow for a better understanding of various physiological responses in marine crustaceans induced by a sudden drop in salinity.

## Additional file

Additional file 1: **Table S1.** The gene-specific primers used in this study, **Table S2.** Clean reads quality metrics from the gill of *S. paramamosain,* **Table S3.** Quality metrics of unigenes from the gill of *S. paramamosain,* **Table S4.** DEGs annotation, **Table S5.** Human Diseases pathways and DEGs involved, **Table S6.** Environmental Information Processing pathways and DEGs involved, **Table S7.** Genetic Information Processing pathways and DEGs involved, **Table S8.** Metabolism pathways and DEGs involved, **Figure S1.** Distribution of base quality on clean reads from the gill of *S. paramamosain*, **Figure S2.** Venn diagram between NR, KOG, KEGG, Swissprot and Interpro. **Figure S3.** The distribution of DEGs in GO analysis, **Figure S4.** Validity of DEGs in Transcriptomic data. (DOCX 1505 kb)

## Abbreviations

GO: Gene Ontology
KEGG: Kyoto Encyclopedia of Genes and Genomes
LS: Low salinity
S. *paramamosain*: *Scylla paramamosain*
sqRT-PCR: semiquantitative reverse-transcription PCR
