# Supplementary material for: Transcriptomic analysis of adaptive mechanisms in response to sudden salinity drop in the mud crab, Scylla paramamosain
Source: BMC Genomics. 2018 May 31;19:421. doi: 10.1186/s12864-018-4803-x (PMC5984308; doi:10.1186/s12864-018-4803-x)
Supplement: Supplementary file 1 — Table S1. The gene-specific primers used in this study, Table S2. Clean reads quality metrics from the gill of S. paramamosain, Table S3. Quality metrics of unigenes from the gill of S. paramamosain, Table S4. DEGs annotation, Table S5. Human Diseases pathways and DEGs involved, Table S6. Environmental Information Processing pathways and DEGs involved, Table S7. Genetic Information Processing pathways and DEGs involved, Table S8. Metabolism pathways and DEGs involved, Figure S1. Distribution of base quality on clean reads from the gill of S. paramamosain, Figure S2. Venn diagram between NR, KOG, KEGG, Swissprot and Interpro. Figure S3. The distribution of DEGs in GO analysis, Figure S4. Validity of DEGs in Transcriptomic data. (DOCX 1505 kb) [file 12864_2018_4803_MOESM1_ESM.docx]

**Additional files**

**Table S1 The gene-specific primers used in this study**

| Functions | Primers | Sequences |
| --- | --- | --- |
| sqRT-PCR | CL1096.Contig1_All | S- AGTTCCGTCACCACACCG |
|  |  | A- TCACCGCAAGCATCCTCT |
|  | CL2951.Contig2_All | S- GCGAGGACGACGACTTGTTT |
|  |  | A- TATCTGTCAGGGCTGGGTTT |
|  | Unigene41750_All | S- GACGGCGAGGGCTGGAGTTT |
|  |  | A- CCCGGCGTTGGGAGGATAGT |
|  | CL4861.Contig2_All | S- ACGAGGGCAAGTTTTGGTGT |
|  |  | A- GCGTCCTGCTGTGTGATGAG |
|  | CL979.Contig3_All | S- GTCAAACACTGTCGGCGGAGG |
|  |  | A- GGAGTAGGAAGGGAGGAACCA |
|  | CL41.Contig2_All | S- AGGTGATGGAGGGCAGTG |
|  |  | A- GCCGAGGAGGAAAAAGGT |
|  | CL4395.Contig1_All | S- GAGACCACAGCCCGACAACC |
|  |  | A- CTTCCTCCTTTCACACACCG |
|  | CL3482.Contig1_All | S- CGGCAAGCATTCCTCACCA |
|  |  | A- AGGCACTCAAAAGACCCCA |
|  | CL94.Contig5_All | S- CCGTCTCCTCCTACTACCCTC |
|  |  | A- GCAGAACTCCCACAGCCCCAT |
|  | CL6558.Contig1_All | S- TGCTCAACCTCCACTACGG |
|  |  | A- CAGCCACCGACAGATTTCAC |
|  | Actin (beta-actin) | S- TCACACACTGTCCCCATCTACG |
|  |  | A- ACCACGCTCGGTCAGGATTTTC |
|  | 18S rRNA | S- AAGTCGTAACAAGGTTTCCGTAG  A- GCGACCACCCACTATTTGTATT |

sqRT-PCR, semiquantitative reverse-transcription PCR.

**Table S2** **Clean reads quality metrics from the gill of *S. paramamosain***

| **Sample** | **Total Raw Reads**  **(Mb)** | **Total Clean Reads**  **(Mb)** | **Total Clean Bases**  **(Gb)** | | **Clean**  **Reads Q20(%)** | | **Clean Reads Q30(%)** | | **Clean Reads Ratio(%)** | |
| --- | --- | --- | --- | --- | --- | --- | --- | --- | --- | --- |
| CK_1 | 77.20 | 66.59 | 6.66 | 96.17 | | 87.78 | | 86.25 | |  |
| CK_2 | 74.66 | 66.04 | 6.60 | | 96.40 | | 88.33 | | 88.46 | |
| CK_3 | 77.16 | 66.38 | 6.64 | | 96.25 | | 88.01 | | 86.03 | |
| LS_1 | 74.67 | 65.01 | 6.50 | | 96.26 | | 88.01 | | 87.07 | |
| LS_2 | 77.15 | 66.26 | 6.63 | | 96.27 | | 88.14 | | 85.89 | |
| LS_3 | 77.20 | 65.68 | 6.57 | | 95.97 | | 87.29 | | 85.08 | |

**Sample**: Sample name; **Total Raw Reads(Mb)**: The reads amount before filtering; **Total Clean Reads(Mb)**:The reads amount after filtering; **Total Clean Bases(Gb)**: The total base amount after filtering; **Clean Reads Q20(%)**: The rate of bases which quality is greater than 20 value in clean reads; **Clean Reads Q30(%)**: The rate of bases which quality is greater than 30 value in clean reads; **Clean Reads Ratio(%)**: The ratio of the amount of clean reads.

**Table S3 Quality metrics of unigenes from the gill of *S. paramamosain***

| **Sample** | **Total Number** | **Total Length（bp）** | **Mean Length（bp）** | **N50** | **N70** | **N90** | **GC(%)** |
| --- | --- | --- | --- | --- | --- | --- | --- |
| CK_1 | 64,232 | 43,863,379 | 682 | 1,141 | 545 | 269 | 45.84 |
| CK_2 | 68,175 | 46,509,780 | 682 | 1,131 | 545 | 269 | 45.93 |
| CK_3 | 66,566 | 42,286,611 | 635 | 977 | 484 | 261 | 46.13 |
| LS_1 | 60,026 | 40,113,391 | 668 | 1,073 | 529 | 268 | 46.12 |
| LS_2 | 66,023 | 41,273,706 | 625 | 951 | 475 | 258 | 46.31 |
| LS_3 | 57,391 | 48,651,699 | 847 | 1,553 | 757 | 311 | 47.18 |
| All-Unigene | 105,171 | 83,038,051 | 789 | 1,512 | 686 | 285 | 46.25 |

**Sample**: Sample name; **Total Number**: The total number of Unigenes; **Total Length**: The read length of Unigenes; **Mean Length**: The average length of Unigenes; **N50**: The N50 length is used to determine the assembly continuity, the higher the better. N50 is a weighted median statistic that 50% of the total length is contained in transcripts that are equal to or larger than this value. **N70**: Similar to N50. **N90**: Similar to N50. **GC(%)**: the percentage of G and C bases in all Unigenes.

**Table S4 DEGs annotation**

| Gene ID | Nr description database | Fold change |
| --- | --- | --- |
| CL3353.Contig2 | BUD13 homolog[Ceratotherium simum simum] | 90.13 |
| CL90.Contig3 | serine/threonine-protein kinase [Nasonia vitripennis] | 71.65 |
| CL2951.Contig2 | beta-1,4-N-acetylgalactosaminyltransferase [Limulus polyphemus] | 67.99 |
| CL1287.Contig2 | tryptophan--tRNA ligase [Strongylocentrotus purpuratus] | 39.16 |
| CL58.Contig6 | tafazzin [Lepisosteus oculatus] | 31.89 |
| Unigene28996 | uncharacterized protein LOC105692508 [Athalia rosae] | 27.07 |
| Unigene19757 | protein AATF-like [Crassostrea gigas] | 24.98 |
| Unigene26911 | transmembrane protein [Tetrahymena thermophila SB210] | 23.23 |
| CL5218.Contig9 | protein NDRG3 [Tribolium castaneum] | 19.98 |
| Unigene41655 | alpha-N-acetylneuraminide alpha-2,8-sialyltransferase [Monodelphis domestica] | 19.62 |
| Unigene10157 | Ribosomal protein S5 domain 2-type fold [Pseudocohnilembus persalinus] | 18.37 |
| Unigene19756 | protein AATF-like [Crassostrea gigas] | 17.83 |
| Unigene2652 | unnamed protein product [Oncorhynchus mykiss] | 17.29 |
| Unigene11185 | inositol-trisphosphate 3-kinase A [Cimex lectularius] | 16.89 |
| Unigene45614 | cytochrome c [Marsupenaeus japonicus] | 16.41 |
| CL1683.Contig5 | Polyubiquitin-C [Thelohanellus kitauei] | 15.98 |
| CL2023.Contig3 | uncharacterized protein LOC101853064 [Aplysia californica] | 15.93 |
| CL1683.Contig3 | Polyubiquitin-C [Thelohanellus kitauei] | 15.92 |
| CL3751.Contig1 | nucleoside diphosphate kinase 7-like [Crassostrea gigas] | 15.88 |
| CL4600.Contig3 | multidrug resistance-associated protein [Athalia rosae] | 15.46 |
| CL401.Contig2 | protein furry [Ceratosolen solmsi marchali] | 14.28 |
| Unigene44405 | putative RNA-directed DNA polymerase from transposon BS [Exaiptasia pallida] | 13.58 |
| Unigene36256 | hypothetical protein [Paramecium tetraurelia strain d4-2] | 13.33 |
| Unigene25468 | mucin-2-like, partial [Salmo salar] | 13.22 |
| CL4852.Contig1 | vegetative cell wall protein gp1-like [Callorhinchus milii] | 12.88 |
| CL1096.Contig3 | E3 ubiquitin-protein ligase AMFR-like [Limulus polyphemus] | 12.63 |
| Unigene40670 | zonadhesin-like [Xenopus tropicalis] | 12.25 |
| Unigene9163 | EF-hand calcium-binding domain-containing protein 2, partial [Cariama cristata] | 12.10 |
| Unigene9170 | peptidylprolyl isomerase [Paramecium primaurelia] | 12.04 |
| Unigene38622 | sodium/hydrogen exchanger [Carcinus maenas] | 11.89 |
| CL1565.Contig2 | von willebrand factor type A (VWA) domain [Tetrahymena thermophila SB210] SB210] | 11.69 |
| CL1611.Contig1 | protein outspread [Polistes dominula] | 11.10 |
| Unigene17070 | sodium/hydrogen exchanger [Carcinus maenas] | 11.08 |
| Unigene18402 | von Willebrand factor type A domain-containing protein [Naegleria gruberi] | 11.05 |
| Unigene35344 | hsp70 [Ichthyophthirius multifiliis] | 10.95 |
| CL5876.Contig1 | sodium- and chloride-dependent glycine transporter 2-like [Limulus polyphemus] | 10.68 |
| Unigene11857 | ER-type hsp70 [Paramecium tetraurelia] | 10.50 |
| Unigene13067 | Histone acetyltransferase MYST3 [Macaca fascicularis] | 10.43 |
| Unigene36931 | zinc ZZ type family protein, putative [Ichthyophthirius multifiliis] | 10.17 |
| CL5876.Contig2 | sodium- and chloride-dependent glycine transporter 2-like [Limulus polyphemus] | 9.94 |
| Unigene56486 | gEBNA-2 nuclear protein [Brugia malayi] | 9.51 |
| Unigene18314 | ubiquitin-activating enzyme E1 [Tetrahymena thermophila SB210] | 9.37 |
| CL1386.Contig1 | glycine-rich cell wall structural protein 1.8-like [Acyrthosiphon pisum] | 9.31 |
| Unigene40814 | Cell surface glycoprotein 1 [Tupaia chinensis] | 9.23 |
| Unigene44862 | keratin, type I cytoskeletal 9 [Marmota marmota marmota] | 8.94 |
| Unigene41750 | gamma-interferon induced thiol reductase GILT1 [Scylla paramamosain] | 8.62 |
| Unigene30364 | putative protein TPRXL [Callorhinchus milii] | 8.55 |
| Unigene36395 | Heterogeneous nuclear ribonucleoprotein K [Zootermopsis nevadensis] | 8.42 |
| CL2988.Contig2 | coiled-coil domain-containing protein 50 [Tribolium castaneum] | 8.24 |
| Unigene23731 | unnamed protein product [Oncorhynchus mykiss] | 7.91 |
| Unigene12705 | sodium-hydrogen exchanger 3 [Cherax cainii] | 7.86 |
| CL831.Contig2 | UNKNOWN [Stylonychia lemnae] | 7.78 |
| CL4852.Contig3 | basic proline-rich protein-like [Halyomorpha halys] | 7.59 |
| Unigene4929 | betaGal beta-1,3-N-acetylglucosaminyltransferase 9 [Stomoxys calcitrans] | 7.51 |
| Unigene19465 | zinc ZZ type family protein, putative [Ichthyophthirius multifiliis] | 7.42 |
| CL869.Contig3 | SEC14-like protein 1 [Zootermopsis nevadensis] | 7.34 |
| CL452.Contig3 | collagen alpha-5(IV) chain [Acanthisitta chloris] | 7.26 |
| CL3543.Contig1 | conserved hypothetical protein [Leishmania major strain Friedlin] | 7.03 |
| CL2638.Contig2 | mucin-2-like [Camelus ferus] | 6.74 |
| Unigene1603 | MD-2-related lipid-recognition protein-like, partial [Nasonia vitripennis] | 6.74 |
| CL3543.Contig2 | conserved hypothetical protein [Leishmania major strain Friedlin] | 6.55 |
| CL2638.Contig1 | AN1-type zinc finger and ubiquitin domain-containing protein 1 [Crassostrea gigas] | 6.52 |
| Unigene21158 | hypothetical protein ENH_00000920 [Eimeria necatrix] | 6.52 |
| Unigene21528 | hypothetical protein T265_09854 [Opisthorchis viverrini] | 6.41 |
| CL4861.Contig2 | ALF1 [Scylla paramamosain] | 6.41 |
| CL3933.Contig2 | collagen alpha-5(IV) chain-like [Diaphorina citri] | 6.38 |
| Unigene17519 | hypothetical protein OCBIM_22022112mg, partial [Octopus bimaculoides] | 6.32 |
| CL869.Contig2 | SEC14-like protein 1 [Zootermopsis nevadensis] | 6.31 |
| CL3443.Contig1 | membrane progestin receptor gamma-B-like [Crassostrea gigas] | 6.06 |
| CL1389.Contig1 | nuclear receptor coactivator [Monomorium pharaonis] | 5.96 |
| Unigene6397 | Prestin [Zootermopsis nevadensis] | 5.84 |
| Unigene111 | 6-phosphofructo-2-kinase/fructose-2,6-bisphosphatase[Tribolium castaneum] | 5.75 |
| CL5283.Contig2 | Sialidase [Melipona quadrifasciata] | 5.55 |
| CL1096.Contig5 | E3 ubiquitin-protein ligase AMFR-like[Limulus polyphemus] | 5.48 |
| Unigene6859 | proteophosphoglycan ppg4 [Leishmania infantum JPCM5] | 5.48 |
| Unigene19965 | X-linked retinitis pigmentosa GTPase regulator-like [Colobus angolensis palliatus] | 5.39 |
| Unigene25463 | uncharacterized protein LOC105280197 [Cerapachys biroi] | 5.34 |
| Unigene29924 | Phosphoenolpyruvate carboxykinase, related [Eimeria necatrix] | 5.15 |
| Unigene113 | 6-phosphofructo-2-kinase/fructose-2,6-bisphosphatase-like[Cimex lectularius] | 5.15 |
| CL4579.Contig2 | ribonuclease P protein subunit p25-like protein [Esox lucius] | 4.90 |
| CL1003.Contig1 | Putative uncharacterized protein FLJ37770, partial [Camponotus floridanus] | 4.79 |
| CL1106.Contig1 | WD repeat and HMG-box DNA-binding protein[Salmo salar] | 4.68 |
| CL850.Contig2 | carbohydrate sulfotransferase 4-like[Wasmannia auropunctata] | 4.68 |
| Unigene112 | 6-phosphofructo-2-kinase/fructose-2,6-bisphosphatase[Tribolium castaneum] | 4.63 |
| Unigene15693 | carbonic anhydrase [Cherax cainii] | 4.63 |
| Unigene17270 | 6-phosphofructo-2-kinase/fructose-2,6-bisphosphatase-like[Cimex lectularius] | 4.57 |
| CL6217.Contig3 | uncharacterized protein LOC100116049 [Nasonia vitripennis] | 4.37 |
| CL979.Contig1 | Solute carrier family 2, facilitated glucose transporter member 3 [Zootermopsis nevadensis] | 4.29 |
| Unigene31721 | 6-phosphofructo-2-kinase/fructose-2,6-bisphosphatase-like[Cimex lectularius] | 4.27 |
| Unigene5252 | uncharacterized protein LOC107271421 [Cephus cinctus] | 4.21 |
| CL979.Contig3 | solute carrier family 2, facilitated glucose transporter member 1-like isoform X1 [Polistes dominula | 4.19 |
| CL5072.Contig1 | magnesium transporter NIPA2-like[Crassostrea gigas] | 4.14 |
| CL6075.Contig1 | T. brucei spp.-specific protein [Trypanosoma brucei gambiense DAL972] | 3.94 |
| Unigene35501 | unnamed protein product [Oncorhynchus mykiss] | 3.87 |
| CL6613.Contig2 | uncharacterized protein Dyak_GE21163, isoform B [Drosophila yakuba] | 3.81 |
| CL2022.Contig3 | uncharacterized protein Dere_GG26582 [Drosophila erecta] | 3.81 |
| CL6599.Contig1 | protein FAM43A-like [Limulus polyphemus] | 3.80 |
| Unigene10665 | Halomucin [Trichinella patagoniensis] | 3.76 |
| CL6154.Contig2 | collagen alpha-1(XXVII) chain [Pongo abelii] | 3.74 |
| Unigene15457 | unnamed protein product [Oncorhynchus mykiss] | 3.42 |
| Unigene15456 | unnamed protein product [Oncorhynchus mykiss] | 3.22 |
| Unigene12149 | cystatin A precursor [Portunus trituberculatus] | 3.06 |
| Unigene11331 | sodium/potassium-transporting ATPase subunit alpha-like [Limulus polyphemus] | 3.03 |
| Unigene44 | multiple epidermal growth factor-like domains protein 10 [Diachasma alloeum] | 3.02 |
| CL6613.Contig4 | transmembrane protein 184B [Linepithema humile] | 3.02 |
| CL6613.Contig1 | transmembrane protein 184B [Linepithema humile] | 2.89 |
| Unigene15443 | chloride channel-a, isoform D [Drosophila melanogaster] | 2.81 |
| Unigene10671 | serine proteinase inhibitor-3 [Eriocheir sinensis] | 2.63 |
| CL94.Contig5 | uncharacterized protein LOC107039269 [Diachasma alloeum] | -34.61 |
| CL643.Contig10 | kinesin light chain[Wasmannia auropunctata] | -34.03 |
| CL5376.Contig4 | aquaporin-12 [Plutella xylostella] | -27.99 |
| CL643.Contig9 | gi\|939644922\|ref\|XP_014272515.1\|/4.7e-237/PREDICTED: kinesin light chain isoform X5 [Halyomorpha halys] | -18.95 |
| CL4395.Contig1 | mannose-binding protein [Portunus pelagicus] | -17.96 |
| CL1287.Contig1 | tryptophan--tRNA ligase, cytoplasmic [Strongylocentrotus purpuratus] | -17.84 |
| CL58.Contig5 | tafazzin [Lepisosteus oculatus] | -17.32 |
| Unigene44550 | DD5 [Marsupenaeus japonicus] | -16.96 |
| CL5331.Contig1 | putative articulin p60 [Rimicaris exoculata] | -16.55 |
| CL3399.Contig2 | glutamine synthetase [Fenneropenaeus chinensis] | -16.14 |
| CL234.Contig2 | uncharacterized protein Dana_GF18486 [Drosophila ananassae] | -12.80 |
| CL643.Contig16 | kinesin light chain isoform X4 [Wasmannia auropunctata] | -12.17 |
| CL3389.Contig2 | hypothetical protein DAPPUDRAFT_305543 [Daphnia pulex] | -11.61 |
| CL4944.Contig1 | putative salivary protein [Culicoides sonorensis] | -11.36 |
| CL4481.Contig6 | von Willebrand factor A domain-containing protein 5A-like [Monodelphis domestica] | -11.26 |
| CL3482.Contig1 | Synaptic vesicle 2-related protein [Zootermopsis nevadensis] | -10.30 |
| CL5707.Contig3 | Sialidase [Toxocara canis] | -7.33 |
| CL17.Contig2 | DNA-directed RNA polymerase II subunit RPB1 [Melipona quadrifasciata] | -5.79 |
| Unigene10578 | uncharacterized protein LOC100877037 [Megachile rotundata] | -5.30 |
| Unigene5126 | protein takeout-like [Cephus cinctus] | -5.27 |
| Unigene38779 | GH19320 [Drosophila grimshawi] | -5.17 |
| CL5287.Contig1 | uncharacterized protein Dana_GF17078 [Drosophila ananassae] | -4.67 |
| CL358.Contig1 | unnamed protein product, partial [Oncorhynchus mykiss] | -4.58 |
| Unigene44161 | serine/threonine-protein phosphatase alpha-3 isoform-like [Limulus polyphemus] | -3.63 |
| CL6558.Contig1 | macroglobulin complement-related 1 [Scolopendra subspinipes japonica] | -3.57 |
| CL401.Contig12 | protein furry [Copidosoma floridanum] | -3.55 |
| CL41.Contig2 | urea transporter 2-like [Cerapachys biroi] | -2.75 |

**Fold Change >= 2.00 and Adjusted Pvalue <= 0.05. “-” showed down-regulate gene**.

**Table S5 Human Diseases pathways and DEGs involved.**

| Pathway | DEGs (47) | Pathway ID | Level 2 |
| --- | --- | --- | --- |
| [Platinum drug resistance](file:///E:\论文撰写\青蟹低盐适应性\图\Tabble.%202\通路基因筛选.xlsx#RANGE!gene68) | Unigene45614 | ko01524 | Antineoplastic resistance |
| [EGFR tyrosine kinase inhibitor resistance](file:///E:\论文撰写\青蟹低盐适应性\图\Tabble.%202\通路基因筛选.xlsx#RANGE!gene120) | Unigene32292, Unigene2652 | ko01521 | Antineoplastic resistance |
| [Central carbon metabolism in cancer](file:///E:\论文撰写\青蟹低盐适应性\图\Tabble.%202\通路基因筛选.xlsx#RANGE!gene24) | Unigene33785, CL979.Contig3, CL979.Contig1 | ko05230 | Cancers: Overview |
| [Pathways in cancer](file:///E:\论文撰写\青蟹低盐适应性\图\Tabble.%202\通路基因筛选.xlsx#RANGE!gene25) | Unigene10157, Unigene33785, Unigene27634, CL6599.Contig1, CL979.Contig3, CL452.Contig3, Unigene45614, CL979.Contig1, CL1386.Contig1, Unigene13067, Unigene44, CL3933.Contig2 | ko05200 | Cancers: Overview |
| [MicroRNAs in cancer](file:///E:\论文撰写\青蟹低盐适应性\图\Tabble.%202\通路基因筛选.xlsx#RANGE!gene28) | Unigene32292, Unigene35501, CL3443.Contig1, CL4600.Contig3, Unigene35484, Unigene36212, Unigene36395, Unigene44 | ko05206 | Cancers: Overview |
| [Viral carcinogenesis](file:///E:\论文撰写\青蟹低盐适应性\图\Tabble.%202\通路基因筛选.xlsx#RANGE!gene137) | Unigene36395 | ko05203 | Cancers: Overview |
| [Transcriptional misregulation in cancer](file:///E:\论文撰写\青蟹低盐适应性\图\Tabble.%202\通路基因筛选.xlsx#RANGE!gene140) | Unigene41655, Unigene36212, Unigene23731 | ko05202 | Cancers: Overview |
| [Choline metabolism in cancer](file:///E:\论文撰写\青蟹低盐适应性\图\Tabble.%202\通路基因筛选.xlsx#RANGE!gene153) | CL6599.Contig1, CL4852.Contig1 | ko05231 | Cancers: Overview |
| [Proteoglycans in cancer](file:///E:\论文撰写\青蟹低盐适应性\图\Tabble.%202\通路基因筛选.xlsx#RANGE!gene164) | Unigene44161 | ko05205 | Cancers: Overview |
| [Small cell lung cancer](file:///E:\论文撰写\青蟹低盐适应性\图\Tabble.%202\通路基因筛选.xlsx#RANGE!gene26) | CL452.Contig3, Unigene45614, Unigene44, CL3933.Contig2 | ko05222 | Cancers: Specific types |
| [Renal cell carcinoma](file:///E:\论文撰写\青蟹低盐适应性\图\Tabble.%202\通路基因筛选.xlsx#RANGE!gene31) | Unigene33785, CL979.Contig3, CL979.Contig1 | ko05211 | Cancers: Specific types |
| Prostate cancer | Unigene10157, Unigene27634 | ko05215 | Cancers: Specific types |
| [Colorectal cancer](file:///E:\论文撰写\青蟹低盐适应性\图\Tabble.%202\通路基因筛选.xlsx#RANGE!gene54) | Unigene45614 | ko05210 | Cancers: Specific types |
| [Thyroid cancer](file:///E:\论文撰写\青蟹低盐适应性\图\Tabble.%202\通路基因筛选.xlsx#RANGE!gene57) | CL1386.Contig1 | ko05216 | Cancers: Specific types |
| [Pancreatic cancer](file:///E:\论文撰写\青蟹低盐适应性\图\Tabble.%202\通路基因筛选.xlsx#RANGE!gene127) | CL6599.Contig1 | ko05212 | Cancers: Specific types |
| [Viral myocarditis](file:///E:\论文撰写\青蟹低盐适应性\图\Tabble.%202\通路基因筛选.xlsx#RANGE!gene34) | Unigene45614, Unigene44 | ko05416 | Cardiovascular diseases |
| [Arrhythmogenic right ventricular cardiomyopathy (ARVC)](file:///E:\论文撰写\青蟹低盐适应性\图\Tabble.%202\通路基因筛选.xlsx#RANGE!gene117) | Unigene44 | ko05412 | Cardiovascular diseases |
| [Hypertrophic cardiomyopathy (HCM)](file:///E:\论文撰写\青蟹低盐适应性\图\Tabble.%202\通路基因筛选.xlsx#RANGE!gene145) | Unigene44 | ko05410 | Cardiovascular diseases |
| [Dilated cardiomyopathy](file:///E:\论文撰写\青蟹低盐适应性\图\Tabble.%202\通路基因筛选.xlsx#RANGE!gene149) | Unigene44 | ko05414 | Cardiovascular diseases |
| [Insulin resistance](file:///E:\论文撰写\青蟹低盐适应性\图\Tabble.%202\通路基因筛选.xlsx#RANGE!gene20) | Unigene33785, CL979.Contig3, CL979.Contig1, Unigene44161 | ko04931 | Endocrine and metabolic diseases |
| [Non-alcoholic fatty liver disease (NAFLD)](file:///E:\论文撰写\青蟹低盐适应性\图\Tabble.%202\通路基因筛选.xlsx#RANGE!gene65) | CL3443.Contig1, Unigene45614 | ko04932 | Endocrine and metabolic diseases |
| [AGE-RAGE signaling pathway in diabetic complications](file:///E:\论文撰写\青蟹低盐适应性\图\Tabble.%202\通路基因筛选.xlsx#RANGE!gene79) | CL452.Contig3, CL3933.Contig2 | ko04933 | Endocrine and metabolic diseases |
| [Vibrio cholerae infection](file:///E:\论文撰写\青蟹低盐适应性\图\Tabble.%202\通路基因筛选.xlsx#RANGE!gene12) | Unigene19977, CL5331.Contig1, CL6613.Contig1, Unigene21158, CL401.Contig2, Unigene9283, Unigene29924, Unigene13827, CL6613.Contig4, CL6613.Contig2, CL5283.Contig2, Unigene34584, Unigene25468, Unigene19978 | ko05110 | Infectious diseases: Bacterial |
| [Staphylococcus aureus infection](file:///E:\论文撰写\青蟹低盐适应性\图\Tabble.%202\通路基因筛选.xlsx#RANGE!gene21) | Unigene44862, CL1386.Contig1 | ko05150 | Infectious diseases: Bacterial |
| [Legionellosis](file:///E:\论文撰写\青蟹低盐适应性\图\Tabble.%202\通路基因筛选.xlsx#RANGE!gene23) | Unigene35344, CL2349.Contig2, Unigene45614 | ko05134 | Infectious diseases: Bacterial |
| [Salmonella infection](file:///E:\论文撰写\青蟹低盐适应性\图\Tabble.%202\通路基因筛选.xlsx#RANGE!gene61) | CL643.Contig16, CL643.Contig10, CL4852.Contig1, CL643.Contig9 | ko05132 | Infectious diseases: Bacterial |
| [Pathogenic Escherichia coli infection](file:///E:\论文撰写\青蟹低盐适应性\图\Tabble.%202\通路基因筛选.xlsx#RANGE!gene89) | Unigene44862, CL1386.Contig1 | ko05130 | Infectious diseases: Bacterial |
| [Tuberculosis](file:///E:\论文撰写\青蟹低盐适应性\图\Tabble.%202\通路基因筛选.xlsx#RANGE!gene115) | Unigene45614 | ko05152 | Infectious diseases: Bacterial |
| [Shigellosis](file:///E:\论文撰写\青蟹低盐适应性\图\Tabble.%202\通路基因筛选.xlsx#RANGE!gene152) | CL4852.Contig1 | ko05131 | Infectious diseases: Bacterial |
| [Bacterial invasion of epithelial cells](file:///E:\论文撰写\青蟹低盐适应性\图\Tabble.%202\通路基因筛选.xlsx#RANGE!gene155) | CL4852.Contig1 | ko05100 | Infectious diseases: Bacterial |
| [Amoebiasis](file:///E:\论文撰写\青蟹低盐适应性\图\Tabble.%202\通路基因筛选.xlsx#RANGE!gene5) | Unigene19977, CL5331.Contig1, CL6613.Contig1, Unigene21158, CL401.Contig2, Unigene9283, Unigene29924, Unigene13827, CL6613.Contig4, CL452.Contig3, CL6613.Contig2, CL5283.Contig2, Unigene34584, Unigene25468, Unigene44, Unigene19978, CL3933.Contig2 | ko05146 | Infectious diseases: Parasitic |
| [Toxoplasmosis](file:///E:\论文撰写\青蟹低盐适应性\图\Tabble.%202\通路基因筛选.xlsx#RANGE!gene16) | Unigene9170, Unigene35344, Unigene45614, Unigene44 | ko05145 | Infectious diseases: Parasitic |
| [Herpes simplex infection](file:///E:\论文撰写\青蟹低盐适应性\图\Tabble.%202\通路基因筛选.xlsx#RANGE!gene59) | Unigene36395, Unigene45614, CL5283.Contig2, Unigene44161, CL3933.Contig2 | ko05168 | Infectious diseases: Viral |
| [HTLV-I infection](file:///E:\论文撰写\青蟹低盐适应性\图\Tabble.%202\通路基因筛选.xlsx#RANGE!gene74) | Unigene33785, CL979.Contig3, CL979.Contig1 | ko05166 | Infectious diseases: Viral |
| [Influenza A](file:///E:\论文撰写\青蟹低盐适应性\图\Tabble.%202\通路基因筛选.xlsx#RANGE!gene85) | Unigene35344, Unigene45614 | ko05164 | Infectious diseases: Viral |
| [Measles](file:///E:\论文撰写\青蟹低盐适应性\图\Tabble.%202\通路基因筛选.xlsx#RANGE!gene91) | Unigene35344 | ko05162 | Infectious diseases: Viral |
| [Hepatitis B](file:///E:\论文撰写\青蟹低盐适应性\图\Tabble.%202\通路基因筛选.xlsx#RANGE!gene100) | Unigene45614 | ko05161 | Infectious diseases: Viral |
| [Epstein-Barr virus infection](file:///E:\论文撰写\青蟹低盐适应性\图\Tabble.%202\通路基因筛选.xlsx#RANGE!gene135) | Unigene35344, CL5283.Contig2, CL1386.Contig1 | ko05169 | Infectious diseases: Viral |
| [Parkinson's disease](file:///E:\论文撰写\青蟹低盐适应性\图\Tabble.%202\通路基因筛选.xlsx#RANGE!gene29) | Unigene18314, Unigene9170, Unigene45614 | ko05012 | Neurodegenerative diseases |
| Prion diseases | Unigene35344, Unigene11857 | ko05020 | Neurodegenerative diseases |
| [Amyotrophic lateral sclerosis (ALS)](file:///E:\论文撰写\青蟹低盐适应性\图\Tabble.%202\通路基因筛选.xlsx#RANGE!gene95) | Unigene45614 | ko05014 | Neurodegenerative diseases |
| [Huntington's disease](file:///E:\论文撰写\青蟹低盐适应性\图\Tabble.%202\通路基因筛选.xlsx#RANGE!gene130) | Unigene9170, Unigene45614 CL5283.Contig2 | ko05016 | Neurodegenerative diseases |
| [Alzheimer's disease](file:///E:\论文撰写\青蟹低盐适应性\图\Tabble.%202\通路基因筛选.xlsx#RANGE!gene141) | Unigene45614 | ko05010 | Neurodegenerative diseases |
| [Amphetamine addiction](file:///E:\论文撰写\青蟹低盐适应性\图\Tabble.%202\通路基因筛选.xlsx#RANGE!gene78) | Unigene44161 | ko05031 | Substance dependence |
| [Alcoholism](file:///E:\论文撰写\青蟹低盐适应性\图\Tabble.%202\通路基因筛选.xlsx#RANGE!gene106) | Unigene44161 | ko05034 | Substance dependence |

**Table S6 Environmental Information Processing pathways and DEGs involved**

| Pathway | DEGs (40) | Pathway ID | Level 2 |
| --- | --- | --- | --- |
| [ABC transporters](file:///E:\论文撰写\青蟹低盐适应性\图\Tabble.%202\通路基因筛选.xlsx#RANGE!gene72) | CL4600.Contig3 | ko02010 | Membrane transport |
| AMPK signaling pathway | Unigene32292, Unigene111, Unigene35501, Unigene113, CL3443.Contig1, Unigene31721, Unigene112, Unigene17270 | ko04152 | Signal transduction |
| PI3K-Akt signaling pathway | Unigene10157_All, Unigene32292_All, Unigene15456, Unigene35501, Unigene27634, Unigene47891, CL922.Contig5, Unigene5252, Unigene15457, CL922.Contig9, CL452.Contig3, CL922.Contig7, Unigene44, CL3933.Contig2 | ko04151 | Signal transduction |
| HIF-1 signaling pathway | Unigene33785, CL979.Contig3, CL979.Contig1 | ko04066 | Signal transduction |
| [cGMP-PKG signaling pathway](file:///E:\论文撰写\青蟹低盐适应性\图\Tabble.%202\通路基因筛选.xlsx#RANGE!gene62) | CL6217.Contig3, Unigene9170, CL41.Contig2, Unigene11331, Unigene44161 | ko04022 | Signal transduction |
| [Phosphatidylinositol signaling system](file:///E:\论文撰写\青蟹低盐适应性\图\Tabble.%202\通路基因筛选.xlsx#RANGE!gene70) | Unigene11185, CL6013.Contig3, Unigene37174 | ko04070 | Signal transduction |
| [Hedgehog signaling pathway - fly](file:///E:\论文撰写\青蟹低盐适应性\图\Tabble.%202\通路基因筛选.xlsx#RANGE!gene83) | CL234.Contig2 | ko04341 | Signal transduction |
| [Hedgehog signaling pathway](file:///E:\论文撰写\青蟹低盐适应性\图\Tabble.%202\通路基因筛选.xlsx#RANGE!gene88) | CL234.Contig2 | ko04340 | Signal transduction |
| [Sphingolipid signaling pathway](file:///E:\论文撰写\青蟹低盐适应性\图\Tabble.%202\通路基因筛选.xlsx#RANGE!gene99) | CL6599.Contig1, CL4600.Contig3 | ko04071 | Signal transduction |
| [cAMP signaling pathway](file:///E:\论文撰写\青蟹低盐适应性\图\Tabble.%202\通路基因筛选.xlsx#RANGE!gene101) | CL6217.Contig3, CL41.Contig2, CL6599.Contig1, Unigene11331, Unigene44161 | ko04024 | Signal transduction |
| [Notch signaling pathway](file:///E:\论文撰写\青蟹低盐适应性\图\Tabble.%202\通路基因筛选.xlsx#RANGE!gene102) | Unigene21528 | ko04330 | Signal transduction |
| [MAPK signaling pathway - fly](file:///E:\论文撰写\青蟹低盐适应性\图\Tabble.%202\通路基因筛选.xlsx#RANGE!gene110) | CL5305.Contig2, Unigene25316 | ko04013 | Signal transduction |
| [Hippo signaling pathway](file:///E:\论文撰写\青蟹低盐适应性\图\Tabble.%202\通路基因筛选.xlsx#RANGE!gene119) | Unigene44161 | ko04390 | Signal transduction |
| [FoxO signaling pathway](file:///E:\论文撰写\青蟹低盐适应性\图\Tabble.%202\通路基因筛选.xlsx#RANGE!gene121) | CL5287.Contig1 | ko04068 | Signal transduction |
| [MAPK signaling pathway](file:///E:\论文撰写\青蟹低盐适应性\图\Tabble.%202\通路基因筛选.xlsx#RANGE!gene124) | Unigene32292, Unigene35344, CL5287.Contig1 | ko04010 | Signal transduction |
| [Calcium signaling pathway](file:///E:\论文撰写\青蟹低盐适应性\图\Tabble.%202\通路基因筛选.xlsx#RANGE!gene125) | Unigene9170, Unigene11185 | ko04020 | Signal transduction |
| [Wnt signaling pathway](file:///E:\论文撰写\青蟹低盐适应性\图\Tabble.%202\通路基因筛选.xlsx#RANGE!gene126) | CL5287.Contig1 | ko04310 | Signal transduction |
| [mTOR signaling pathway](file:///E:\论文撰写\青蟹低盐适应性\图\Tabble.%202\通路基因筛选.xlsx#RANGE!gene134) | Unigene32292, Unigene35501 | ko04150 | Signal transduction |
| [Hippo signaling pathway - fly](file:///E:\论文撰写\青蟹低盐适应性\图\Tabble.%202\通路基因筛选.xlsx#RANGE!gene139) | Unigene25316 | ko04391 | Signal transduction |
| [Ras signaling pathway](file:///E:\论文撰写\青蟹低盐适应性\图\Tabble.%202\通路基因筛选.xlsx#RANGE!gene158) | Unigene32292, CL6599.Contig1 | ko04014 | Signal transduction |
| [Phospholipase D signaling pathway](file:///E:\论文撰写\青蟹低盐适应性\图\Tabble.%202\通路基因筛选.xlsx#RANGE!gene162) | CL6599.Contig1, Unigene5252 | ko04072 | Signal transduction |
| ECM-receptor interaction | CL3482.Contig1, CL922.Contig5, CL922.Contig9, CL452.Contig3, CL922.Contig7, Unigene44, CL3933.Contig2 | ko04512 | Signaling molecules and interaction |
| [Cell adhesion molecules (CAMs)](file:///E:\论文撰写\青蟹低盐适应性\图\Tabble.%202\通路基因筛选.xlsx#RANGE!gene138) | Unigene15456, Unigene47891, Unigene15457 | ko04514 | Signaling molecules and interaction |
| [Neuroactive ligand-receptor interaction](file:///E:\论文撰写\青蟹低盐适应性\图\Tabble.%202\通路基因筛选.xlsx#RANGE!gene163) | Unigene5252 | ko04080 | Signaling molecules and interaction |

**Table. S7 Genetic Information Processing pathways and DEGs involved**

| Pathway | DEGs (28) | Pathway ID | Level 2 |
| --- | --- | --- | --- |
| Protein processing in endoplasmic reticulum | Unigene10157, Unigene35344, Unigene21158, Unigene27634, Unigene11857, Unigene4333, CL1096.Contig1, CL1096.Contig3, CL1096.Contig5 | ko04141 | Folding, sorting and degradation |
| [Ubiquitin mediated proteolysis](file:///E:\论文撰写\青蟹低盐适应性\图\Tabble.%202\通路基因筛选.xlsx#RANGE!gene46) | Unigene18314, Unigene19465, CL358.Contig1, Unigene47891, Unigene26228 | ko04120 | Folding, sorting and degradation |
| Protein export | Unigene11857 | ko03060 | Folding, sorting and degradation |
| RNA degradation | Unigene70319, CL3389.Contig2 | ko03018 | Folding, sorting and degradation |
| [Spliceosome](file:///E:\论文撰写\青蟹低盐适应性\图\Tabble.%202\通路基因筛选.xlsx#RANGE!gene129) | Unigene35344, Unigene18877, Unigene36395, CL2988.Contig2 | ko03040 | Transcription |
| [RNA polymerase](file:///E:\论文撰写\青蟹低盐适应性\图\Tabble.%202\通路基因筛选.xlsx#RANGE!gene157) | CL5283.Contig2 | ko03020 | Transcription |
| Aminoacyl-tRNA biosynthesis | CL1287.Contig2, CL1287.Contig1 | ko00970 | Translation |
| RNA transport | CL6075.Contig1, CL2349.Contig2, CL4579.Contig2, CL2988.Contig2, CL1386.Contig1 | ko03013 | Translation |
| [mRNA surveillance pathway](file:///E:\论文撰写\青蟹低盐适应性\图\Tabble.%202\通路基因筛选.xlsx#RANGE!gene71) | CL17.Contig2, CL2988.Contig2, Unigene44161 | ko03015 | Translation |
| [Ribosome biogenesis in eukaryotes](file:///E:\论文撰写\青蟹低盐适应性\图\Tabble.%202\通路基因筛选.xlsx#RANGE!gene114) | CL4579.Contig2 | ko03008 | Translation |

**Table. S8 Metabolism pathways and DEGs involved**

| Pathway | DEGs (27) | Pathway ID | Level 2 |
| --- | --- | --- | --- |
| Galactose metabolism | CL2951.Contig2 | ko00052 | Amino acid metabolism |
| Amino sugar and nucleotide sugar metabolism | CL2022.Contig3 | ko00520 | Amino acid metabolism |
| Fatty acid metabolism | CL1003.Contig1 | ko01212 | Amino acid metabolism |
| Biosynthesis of amino acids | CL3399.Contig2 | ko01230 | Amino acid metabolism |
| Arginine biosynthesis | CL3399.Contig2 | ko00220 | Carbohydrate metabolism |
| Nitrogen metabolism | Unigene15693, CL3399.Contig2 | ko00910 | Carbohydrate metabolism |
| Sulfur metabolism | Unigene45614 | ko00920 | Carbohydrate metabolism |
| Glycosaminoglycan biosynthesis - keratan sulfate | CL2951.Contig2 | ko00533 | Carbohydrate metabolism |
| Other types of O-glycan biosynthesis | CL2951.Contig2 | ko00514 | Carbohydrate metabolism |
| Glycosphingolipid biosynthesis - ganglio series | Unigene41655 | ko00604 | Carbohydrate metabolism |
| Lysine degradation | CL1611.Contig1, Unigene10578, CL1003.Contig1 | ko00310 | Energy metabolism |
| Fructose and mannose metabolism | Unigene111, Unigene113, Unigene3172, Unigene112, Unigene17270 | ko00051 | Energy metabolism |
| N-Glycan biosynthesis | CL2951.Contig2 | ko00510 | Energy metabolism |
| Metabolic pathways | Unigene4929, CL6599.Contig1, Unigene41655, Unigene11185, CL3399.Contig2, Unigene10578, CL1003.Contig1, CL3751.Contig1, CL6013.Contig3, CL2951.Contig2, Unigene6639, Unigene45614, CL5283.Contig2, Unigene37174, CL2022.Contig3, CL1422.Contig5, Unigene44 | ko01100 | Global and overview maps |
| Glycerophospholipid metabolism | CL58.Contig6, CL6599.Contig1, Unigene37174, CL58.Contig5 | ko00564 | Global and overview maps |
| Ether lipid metabolism | CL6599.Contig1 | ko00565 | Global and overview maps |
| Retinol metabolism | CL6599.Contig1, CL1422.Contig5 | ko00830 | Global and overview maps |
| Alanine, aspartate and glutamate metabolism | CL3399.Contig2 | ko00250 | Glycan biosynthesis and metabolism |
| Valine, leucine and isoleucine degradation | CL1003.Contig1 | ko00280 | Glycan biosynthesis and metabolism |
| Glyoxylate and dicarboxylate metabolism | CL3399.Contig2 | ko00630 | Glycan biosynthesis and metabolism |
| Inositol phosphate metabolism | Unigene11185, CL6013.Contig3 | ko00562 | Glycan biosynthesis and metabolism |
| Oxidative phosphorylation | Unigene44 | ko00190 | Glycan biosynthesis and metabolism |
| Glycosphingolipid biosynthesis - globo series | Unigene41655, Unigene6639 | ko00603 | Glycan biosynthesis and metabolism |
| Citrate cycle (TCA cycle) | Unigene10578 | ko00020 | Lipid metabolism |
| Carbon metabolism | Unigene10578 | ko01200 | Lipid metabolism |
| Fatty acid degradation | CL1003.Contig1 | ko00071 | Lipid metabolism |
| Glycosphingolipid biosynthesis - lacto and neolacto series | Unigene4929, Unigene41655, CL2951.Contig2, Unigene6639 | ko00601 | Metabolism of cofactors and vitamins |
| Pyrimidine metabolism | CL3751.Contig1, CL5283.Contig2 | ko00240 | Nucleotide metabolism |
| Purine metabolism | Unigene30725, CL3751.Contig1, CL5283.Contig2 | ko00230 | Nucleotide metabolism |

**Table. S9** **Cellular Processes pathways and DEGs involved**

| Pathway | DEGs (22) | Pathway ID | Level 2 |
| --- | --- | --- | --- |
| Apoptosis - multiple species | Unigene45614 | ko04215 | Cell growth and death |
| [p53 signaling pathway](file:///E:\论文撰写\青蟹低盐适应性\图\Tabble.%202\通路基因筛选.xlsx#RANGE!gene67) | Unigene45614 | ko04115 | Cell growth and death |
| [Oocyte meiosis](file:///E:\论文撰写\青蟹低盐适应性\图\Tabble.%202\通路基因筛选.xlsx#RANGE!gene104) | Unigene44161 | ko04114 | Cell growth and death |
| [Apoptosis - fly](file:///E:\论文撰写\青蟹低盐适应性\图\Tabble.%202\通路基因筛选.xlsx#RANGE!gene116) | Unigene45614 | ko04214 | Cell growth and death |
| [Apoptosis](file:///E:\论文撰写\青蟹低盐适应性\图\Tabble.%202\通路基因筛选.xlsx#RANGE!gene128) | Unigene45614 | ko04210 | Cell growth and death |
| [Regulation of actin cytoskeleton](file:///E:\论文撰写\青蟹低盐适应性\图\Tabble.%202\通路基因筛选.xlsx#RANGE!gene156) | CL4852.Contig1, Unigene40814, Unigene44161 | ko04810 | Cell motility |
| Focal adhesion | CL922.Contig5, CL922.Contig9, CL452.Contig3, CL922.Contig7, Unigene44161, Unigene44, CL3933.Contig2 | ko04510 | Cellular community |
| [Adherens junction](file:///E:\论文撰写\青蟹低盐适应性\图\Tabble.%202\通路基因筛选.xlsx#RANGE!gene131) | CL4852.Contig1, CL5287.Contig1 | ko04520 | Cellular community |
| [Tight junction](file:///E:\论文撰写\青蟹低盐适应性\图\Tabble.%202\通路基因筛选.xlsx#RANGE!gene154) | CL1611.Contig1 | ko04530 | Cellular community |
| [Lysosome](file:///E:\论文撰写\青蟹低盐适应性\图\Tabble.%202\通路基因筛选.xlsx#RANGE!gene80) | CL855.Contig1, Unigene1603 | ko04142 | Transport and catabolism |
| [Endocytosis](file:///E:\论文撰写\青蟹低盐适应性\图\Tabble.%202\通路基因筛选.xlsx#RANGE!gene103) | CL3543.Contig1, Unigene35344, CL4481.Contig6, CL358.Contig1, CL6599.Contig1, Unigene47891, CL3543.Contig2, Unigene26228 | ko04144 | Transport and catabolism |


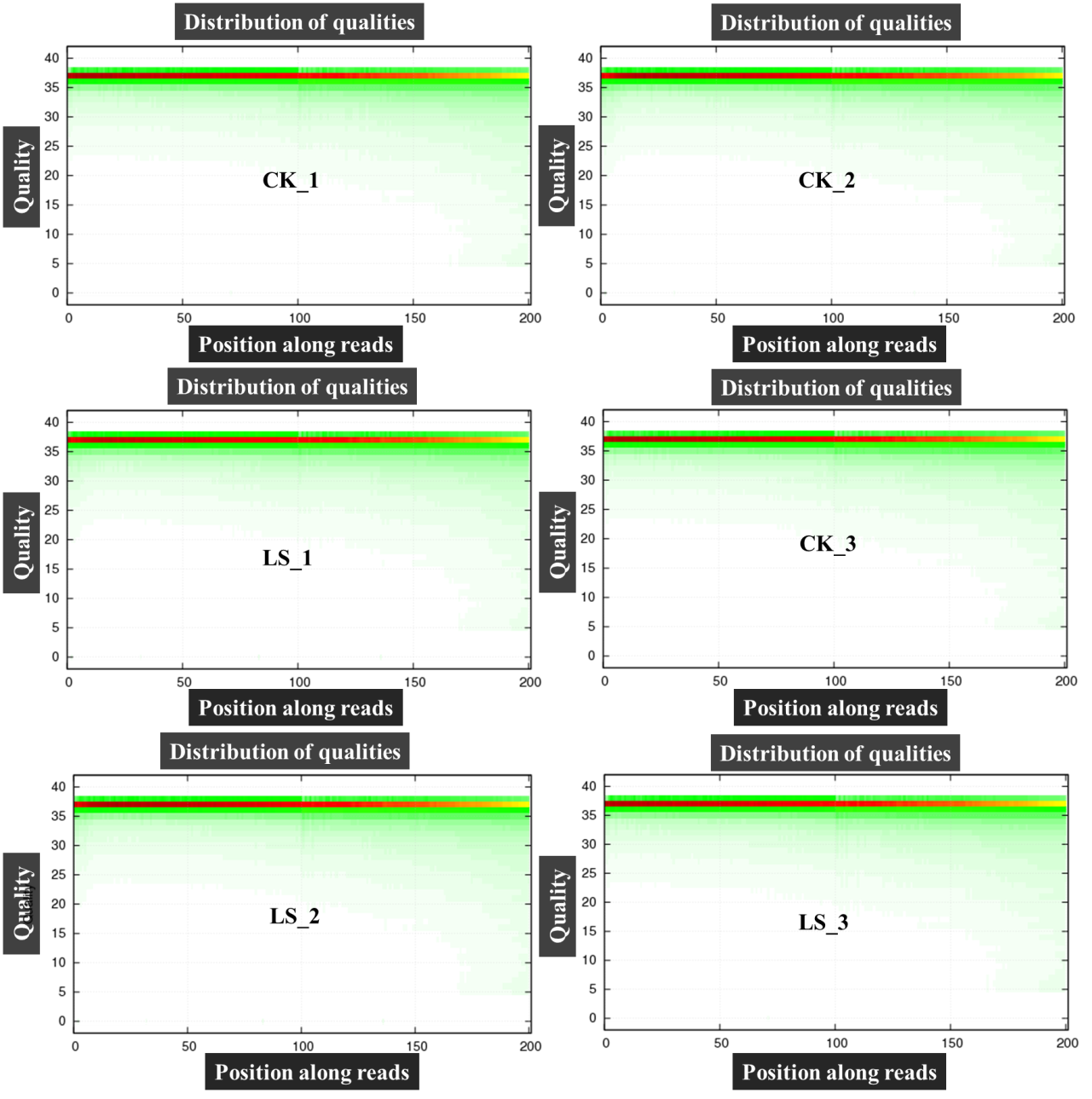


**Fig. S1 Distribution of base quality on clean reads from the gill of *S. paramamosain*.** X axis represents base positions along reads. Y axis represents base quality value. Each dot in the image represents the number of total bases with certain quality value of the corresponding base along reads. Darker dot color means greater base number. If the proportion of the bases with low quality (< 20) is very low, that means the sequencing quality of this lane is good.


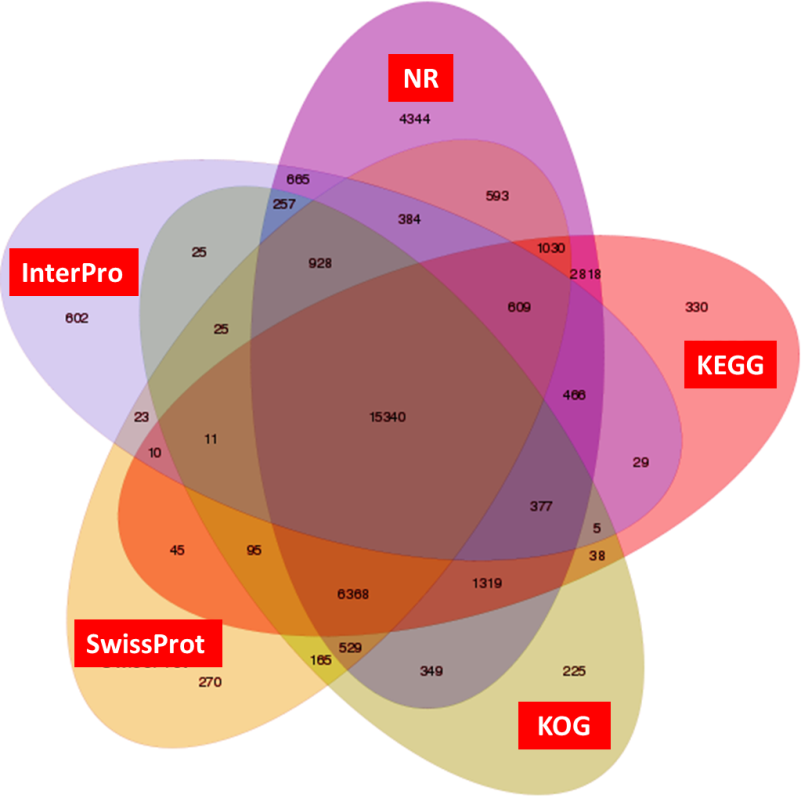


**Fig. S2 Venn diagram between NR, KOG, KEGG, Swissprot and Interpro.** We used Venn diagram to show the annotation result of NR,KOG,KEGG,SwissProt and InterPpro. NT database is the official Nucleic acid database of NCBI and NR database is the official protein

data base of NCBI. GO annotation was also based on data base of NCBI.


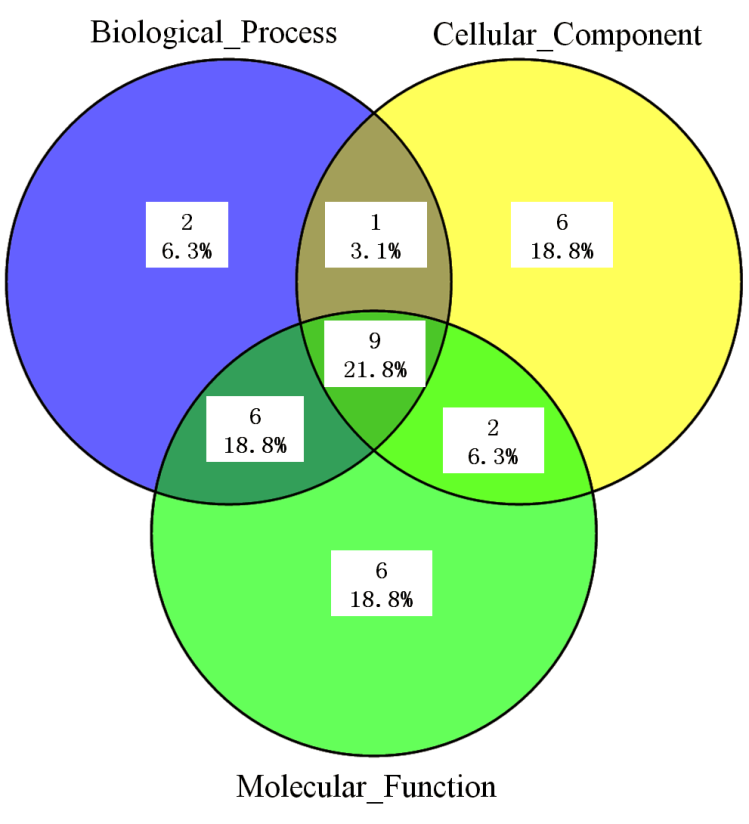


**Fig. S3 The distribution of DEGs in GO analysis**

**
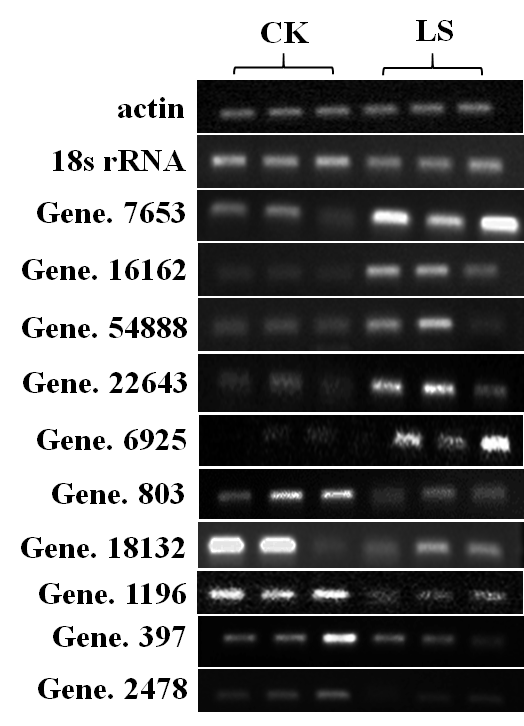
**

**Fig. S4 Validity of DEGs in Transcriptomic data**. Gene. 7653: CL1096.Contig1_All, Gene. 16162: CL2951.Contig2_All, Gene. 54888: Unigene41750_All, Gene. 22643: CL4861.Contig2_All, Gene. 6925: CL979.Contig3_All, Gene. 803: CL94.Contig5_All, Gene. 18132: CL3482.Contig1_All, Gene. 1196:CL4395.Contig1_All, Gene. 397: CL41.Contig2_All, Gene. 2748: CL358.Contig1_All. The *S. paramamosain* beta-actin gene and 18S rRNA gene were selected as the internal control.
